# Emerging stimuli-responsive hydrogels for enhancing chronic wound healing

**DOI:** 10.1039/d5lp00092k

**Published:** 2025-08-27

**Authors:** Maya Yun, Logan Langford, Lewis Russell, Natalie Ndiforamang, Anran Zhang, Wubin Bai

**Affiliations:** a Department of Applied Physical Sciences, University of North Carolina Chapel Hill NC 27514 USA wbai@unc.edu; b Lampe Joint Department of Biomedical Engineering, University of North Carolina Chapel Hill NC 27514 USA; c Department of Environmental Sciences and Engineering, University of North Carolina Chapel Hill NC 27514 USA; d Department of Psychology, University of North Carolina Chapel Hill NC 27514 USA

## Abstract

Stimuli-responsive hydrogels have gained significant attention in wound care due to their ability to adapt to dynamic physiological conditions, making them promising candidates for facilitating chronic wound healing. These hydrogels can respond to both internal and external environmental stimuli such as temperature, pH, reactive oxygen species (ROS), glucose levels, MMP, mechanical forces, magnetism, and ultrasound, enabling precise, on-demand therapeutic interventions through controlled drug release. This responsiveness is governed by reversible changes in their polymer network structure caused by interactions with external stimuli. By creating an optimized environment for wound healing, stimuli-sensitive hydrogels can promote moisture retention, cellular migration, and mechanical flexibility while accelerating critical tissue repair processes like angiogenesis or collagen synthesis. Additionally, incorporating bioactive agents such as antimicrobial compounds, growth factors, and other therapeutically active substances like honey, has further expanded their functionality, though such modifications may be secondary to their inherent stimuli-responsive nature. This review provides a comprehensive overview of recent advancements in stimuli-responsive hydrogels for chronic wound management, highlighting their ability to respond to environmental cues and addressing their potential to enhance healing through the controlled release of therapeutic agents, promotion of hemostasis, and tissue regeneration.

## Abstract 2

Stimuli-responsive hydrogels, activated by biological and external cues, hold promise for controlled drug release, moisture retention, and accelerated tissue regeneration. This review highlights their emerging role in advancing chronic wound therapy.

## Graphical abstract

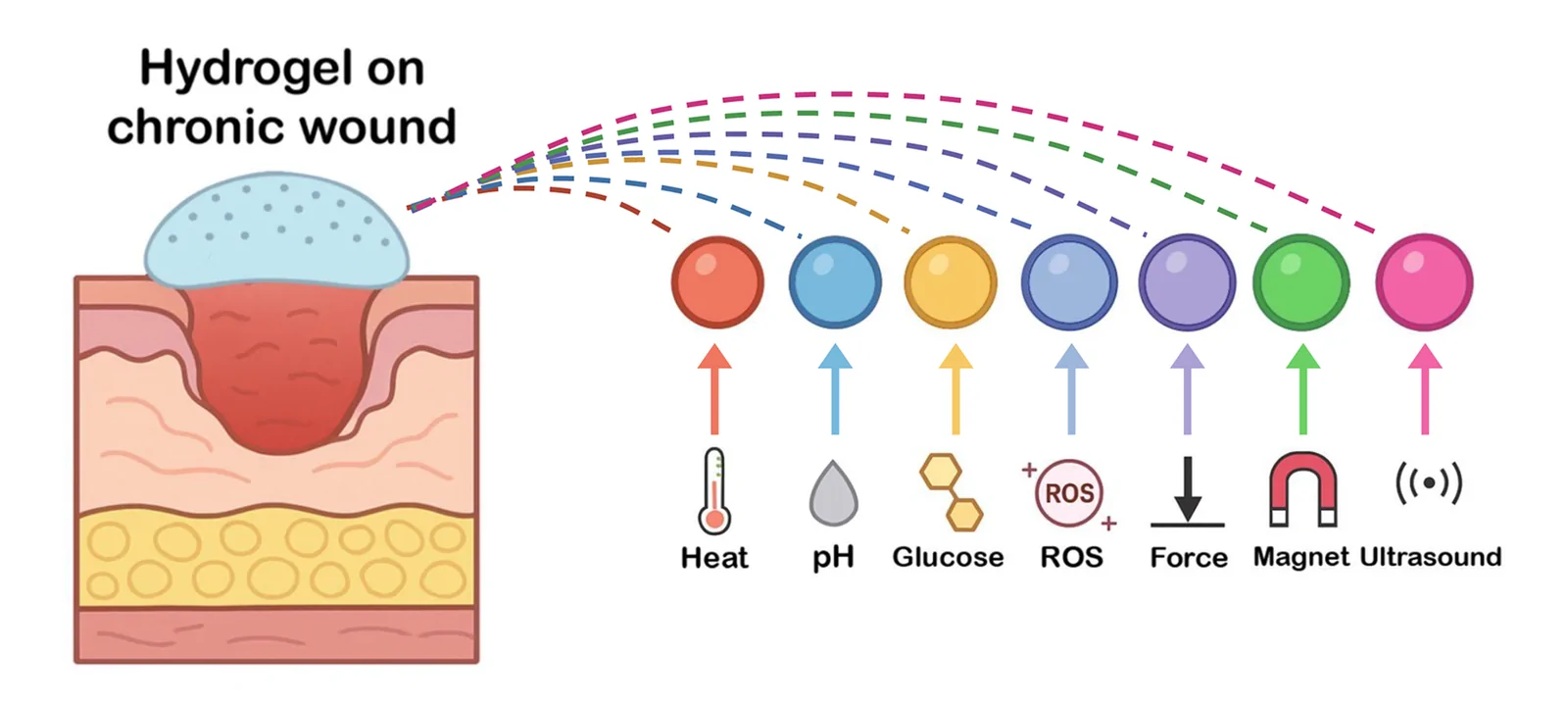

## 1 Introduction

As the largest organ in the human body, the skin plays a multifaceted role in regulating homeostasis and protecting internal organs.^1^ However, through constant exposure to external stimuli, such as in a chronic open wound, skin is particularly vulnerable to infection that can have severe negative and widespread implications for underlying tissue.^2^

Chronic wounds are those that fail to heal within the typical timeframe or frequently recur. These abnormal, nonhealing wounds impact more than 8.2 million people in the US, and were estimated to cost up to $18.7 billion in 2014.^3,4^ Although individuals of various ages can be affected, chronic wounds are most prevalent in elderly populations and encompass conditions such as diabetic foot ulcers, pressure ulcers, and venous leg ulcers. Chronic wounds are most commonly observed in adults with comorbid conditions, including but not limited to diabetes, obesity, and venous or arterial insufficiency, as these factors impair normal wound healing.^5^ Additional factors, such as infections, improper moisture balance, and restricted blood flow to the affected area, can also contribute to the formation of chronic wounds. As of 2018, an estimated 10.5% of the US population live with diabetes, while approximately 42.4% were affected by obesity, as recorded by the Centers for Disease Control and Prevention (CDC) and the World Health Organization (WHO) respectively.^6^ The likelihood of these populations developing chronic wounds would subsequently increase over time; it is estimated that by 2060, over 70 million elderly U.S. individuals would experience persistent chronic wounds. If left untreated, chronic wounds can result in severe complications, including sepsis, limb amputation, and even mortality. The importance of skin as a barrier has facilitated in-depth research efforts to understand the mechanism by which skin wounds can be repaired to restore proper functionality.^7^

Wound dressings play a crucial role in protecting open wounds from external contaminants; however, their effectiveness largely depends on the nature of the wound and the dressings’ properties. Key clinical factors to consider when selecting an appropriate dressing include the amount of wound exudate, skin fragility, and the development of favorable granulation tissue after debridement.^5^ Gauzes are historically the most commonly used for wounds, as they are easily accessible and inexpensive. Although, these dressings have significant downsides; gauze tends to dry out the wound bed, which may cause damage to the new skin barrier during frequent changing, and offers limited protection as a good barrier.^8^ Gauze also becomes ineffective after it is saturated with wound exudate.^8^ Film dressings, while better at maintaining a moisture-retentive environment and protecting the wound from external contamination, have limited swelling capacity, and cannot be used for wounds with exudate or infected wounds, as the nature of the dressing would trap and promote harmful bacterial growth.^9^ Hydrocolloids are beneficial as they are known to promote autolytic debridement. However, the opaque material makes it difficult to visualize any indications of changes underneath, such as from infections; it can also leave behind an unpleasant odor, holding in yellow-brown, gel-like drainage formed from the exudate.^8,10^ Foam dressings are often recommended for wounds that produce more exudate, as these dressings are extremely absorbent. However, they are similar to the disadvantages of hydrocolloids in that they are opaque and can leave malodorous discharges, and they often require a secondary form of dressing as well.^8,10^

An ideal dressing for chronic wounds should not only serve as a protective barrier, but also foster an internal environment conducive to skin tissue regeneration, even in patients with impaired healing conditions. The dressing should be biocompatible, have sufficient absorption of wound exudates, prevent contamination, maintain adequate moisture, and be conformable for the fastest recovery.^11^ The removal should also be painless, consequently causing the least damage to the skin. Hence, improving a dressing's physical and chemical properties is essential for enhanced recovery of chronic wounds. Among the various wound dressing materials studied, stimuli-responsive hydrogels have shown particular promise for enhancing wound healing.^12^ Due to limitations of conventional dressings – such as poor moisture retention, inflexibility, limited absorption, and typically no direct healing properties – innovative materials are needed to overcome the challenges of chronic wound healing. Stimuli-responsive hydrogels show great promise due to their ability to adapt to their environment as the environment fluctuates. These hydrogels are flexible and adjustable biomaterials that allow one to tune various aspects within the hydrogel, such as incorporating different copolymerized materials, or adjusting the critical point of a temperature-sensitive hydrogel for drug release. They create a moist environment that promotes favorable conditions for wound healing, and their hydrophilicity allows for absorption of large quantities of fluid while retaining flexibility and mechanical properties, all while being nontraumatic on the skin of the healing wound.^13^

**Fig. 1:**
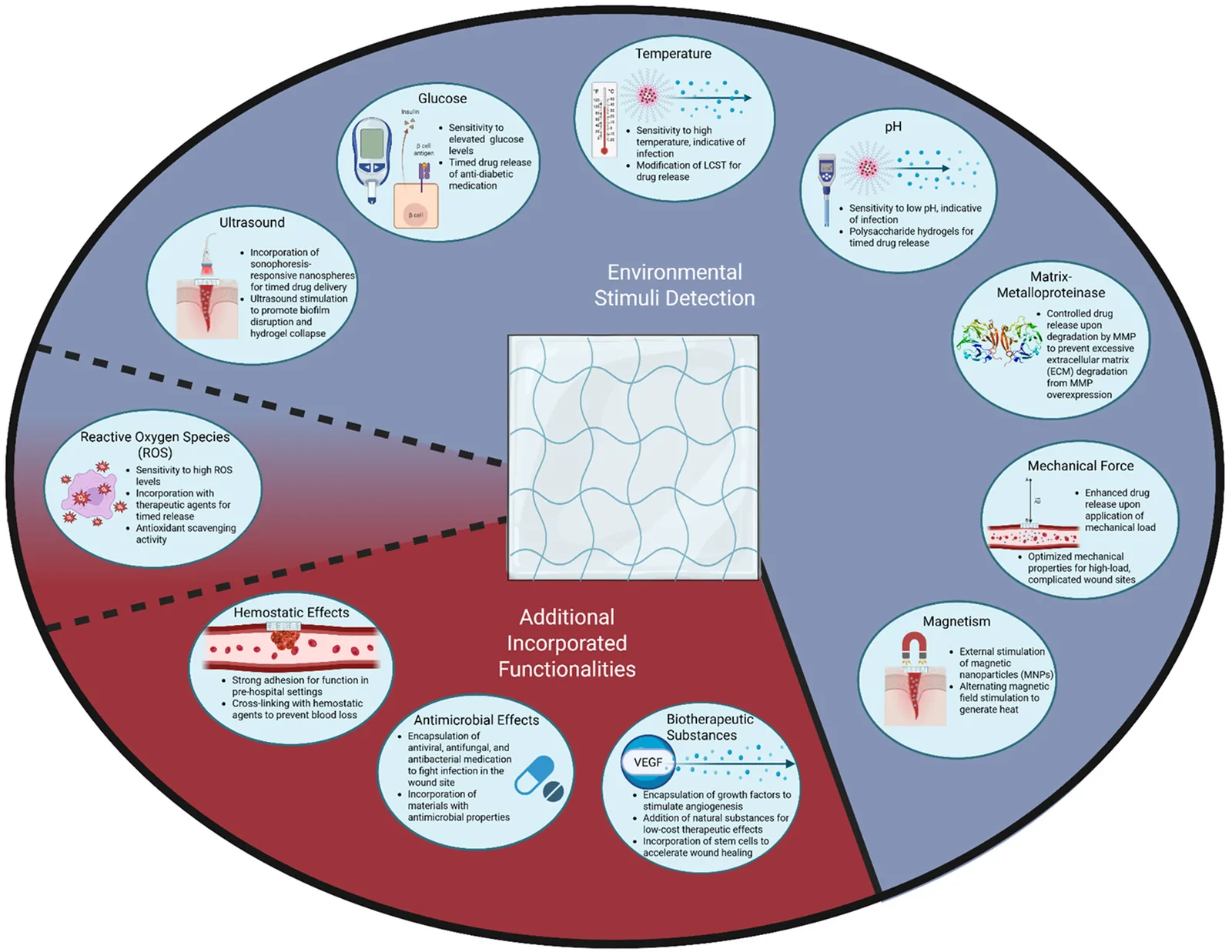
Schematic representation of the functional components and mechanisms of stimuli-responsive hydrogels for wound healing.

Creating hydrogels with certain polymers or materials can provide them the ability to respond to various stimuli, such as heat, pH, glucose, reactive oxygen species (ROS), or matrix-metalloproteinases (MMP). These factors often become imbalanced in chronic wounds, leading to prolonged inflammation and delayed healing. Stimuli-responsive hydrogels can detect these imbalances and release therapeutic agents or change their properties accordingly, effectively promoting a more favorable healing environment for the wound. Developing hydrogel dressing systems that effectively respond to imbalances of these factors can result in improved wound healing in chronic wounds. Hydrogels can also be tuned to respond to applied external stimuli, such as mechanical forces, magnetism, or ultrasound. Building upon previous comprehensive reviews and novel research of the general wound healing process, generic hydrogels, and stimuli-responsive hydrogels, this review provides an updated overview of the general wound healing and the role of hydrogels throughout this process, followed by a discussion of stimuli-responsive hydrogel with a specific focus on recent advances and emerging design strategies, including their response to key environmental factors. Additionally, we examine bioactive and nanocomposite developments and their contributions to hydrogel-based wound healing. An outline of this review is displayed in Fig. 1.

## 2 Normal wound healing process

**Fig. 2:**
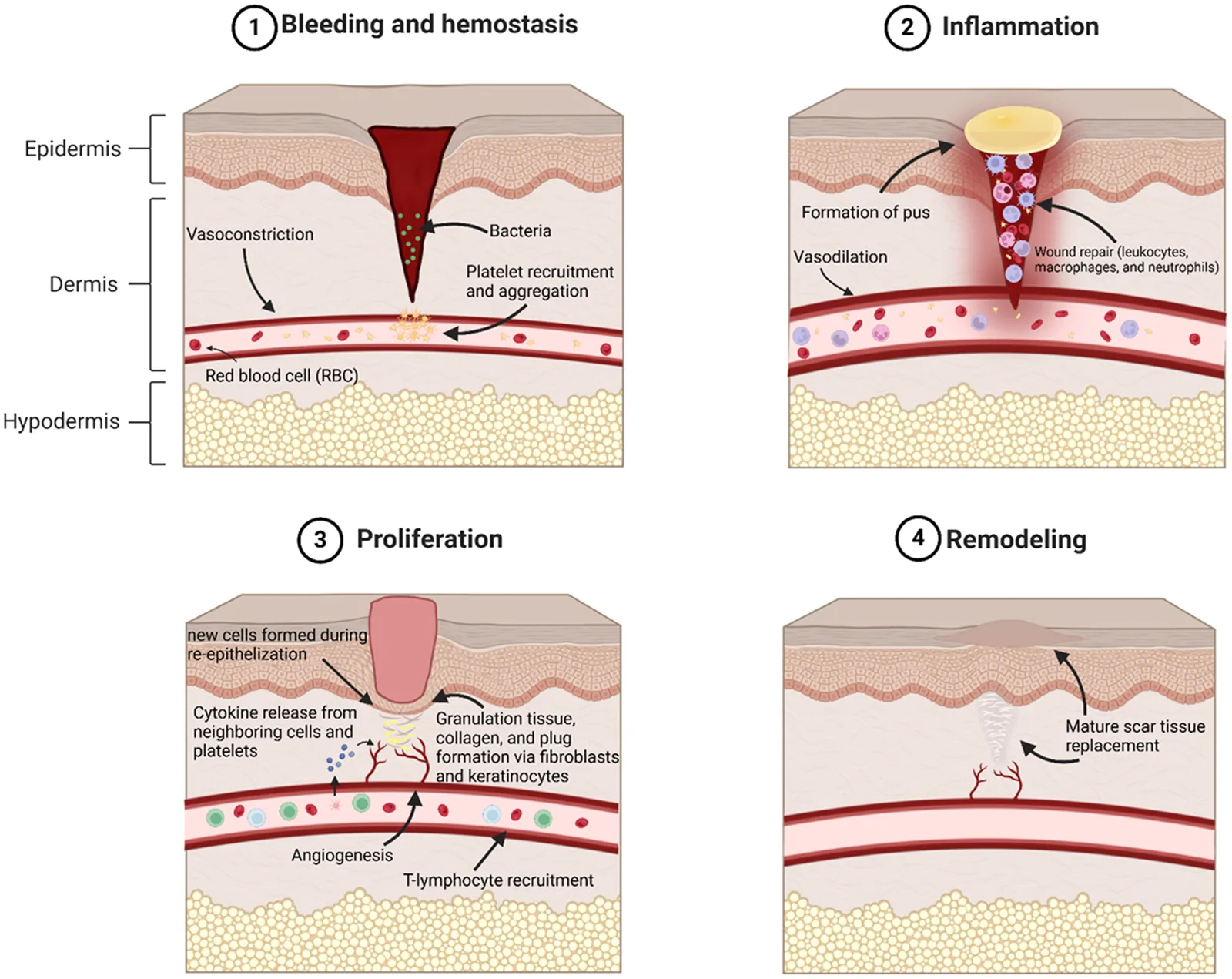
Schematic of normal wound healing phases, including the hemostasis, inflammation, proliferation, and remodeling stages.

The normal process of wound healing can vary depending on the nature and severity of the wound. As outlined by Chhabra *et al.*, the classification of wound healing depends on the amount or area of cell death and disruption to the process.^14^ Therefore, it can be difficult to generally define the steps of wound repair beyond four common stages marked by unique cellular and bio-psychological events: bleeding/hemostasis, inflammation, proliferation, and remodeling. A visual illustration of the wound healing process is shown in Fig. 2.

Immediately following an injury to skin tissue, blood vessels in the dermis may be severed, resulting in bleeding. The severity of bleeding has been found to depend largely on the number of transected vessels and the size or blood flow through each vessels.^15^ For example, transection of the aorta, the largest artery in the human body, has a mortality rate of approximately 90%.^16^ However, the greatest risk associated with smaller, open wounds is infection, as pathogens can bypass the body's protective barrier and spread to internal organs and systems. Roodsari *et al.* found that infection rates for simple hand lacerations treated in the emergency department were upwards of 5%, increasing the patient's risk for life-threatening conditions such as tetanus, sepsis, staph infection, and other severe bacterial or viral diseases.^17^

Hemostasis is a multi-step process initiated by clotting factors and other molecules such as platelets. In order to prevent blood loss, vasoconstriction is triggered by smooth muscle vascular spasms, and platelet aggregation is promoted by the exposed extracellular matrix around the damaged vessel.^18^ While these two actions help to prevent blood loss, they have not yet produced a stable physical barrier for the wound's outer surface, and it is crucial at this point to cover the wound with a dressing to protect the platelet clot, prevent further injury, and maintain a healthy environment for tissue remodeling.^18^

Following hemostasis, the inflammatory stage is characterized by vasodilation, facilitating the infiltration of immune cells such as leukocytes, neutrophils, and macrophages into the extracellular space, where they phagocytose bacteria and necrotic tissue, setting the stage for tissue repair.^19^ As outlined by Hosgood, the accumulation and eventual breakdown of immune cells and fluid manifest as pus. Longer-lasting monocytes are also recruited to the now-cleaned wound site by a cytokine storm released from dying neutrophils, and their proliferation is responsible for the inflamed appearance of wounds before their repair.^19^ In order to transition from inflammation to tissue remodeling and repair, several key events typically occur within the wound. As detailed by Landén *et al.*, macrophages play a crucial role in both promoting and suppressing inflammation at different points, termed the M1–M2 transition. This, in turn, makes macrophages and their secreted mediators popular therapeutic targets due to their high importance in both initiating and terminating inflammation.^20^ If this transition, among other key events, does not occur as planned, proliferation and remodeling can be detrimentally delayed, increasing the risk of chronic wound formation.

Assuming a successful quenching of inflammation within the tissue, the wound healing process continues to the proliferation stage, marked by angiogenesis, or the reformation of vascular networks on the wound site.^7^ Granulation tissue also begins to form near the surface of the wound through a process involving both CD4 and CD8 T-lymphocytes, keratinocytes, and several growth factors and cytokines, such as Th1, Th2, and Th17.^7^ Granulation tissue serves to provide the wound with a protective epithelial layer and a stronger and more versatile plug during the proliferation of underlying tissue and vessels.^21^ Simultaneously, reepithelialization occurs around the wound to stretch and close the lesion, and collagen is reintroduced into the new epithelial tissue from fibroblasts. It is crucial during this stage that the wound remains clean and protected, as any injuries or foreign bodies in the site have been associated with poor chronic wound healing.

In the final stage, known as the remodeling stage, apoptosis of immature collagen and granulation tissue allows for replacement by longer-lasting scar tissue.^21^ Mature collagen fibers are incorporated into the new tissue to increase tensile strength, and inflammation is reduced by apoptosis of excess blood vessels introduced to supply the wound healing process in the inflammatory stage. Through differentiation of fibroblasts into myofibroblasts, scar tissue is eventually contracted, and the extracellular matrix around the tissue thickens, but this process may take years to completely heal.^22^ Furthermore, any further injuries to the region during this stage have been shown to alter levels of collagen within the wound site and lead to long-term scarring.^23^

### 2.1 Chronic wound healing process

Chronic wounds differ in their pattern of healing and are an ongoing struggle in healthcare to promptly and effectively treat. Chronic wounds can result from several diseases with unique pathophysiologies that result in wound conditions unfavorable for successful wound healing.^24^ Consequently, the undesirable conditions within the wound prolong the healing process, increasing the risk of infection and worsening the mobility and quality of life in affected patients.

While the overall stages outlined previously are still present in chronic wound healing, a distinction is often made in the order of these events. Unlike the linear approach to healing that occurs in normal wounds, chronic wounds exhibit a non-linear, or asynchronous, wound healing pattern with overlapping periods of hemostasis, inflammation, remodeling, and cell proliferation. In other words, disruptions at some point during the wound healing process can prolong or cut short certain stages of the wound healing process with potentially detrimental effects to the affected tissue.^25^ The tissue repair and proliferation stages may be disrupted by a prolonged inflammatory state, resulting from an inability of the surrounding tissue to remove inflammatory mediators such as M1 macrophages, tumor necrosis factors (TNF), and ROS.^5^ This extended inflammatory period has been historically difficult to prevent due to diverse etiologies and comorbidities involved in the development of chronic wounds, each with different presentations and potential therapeutic targets. However, the resulting conditions and presentation of these varying pathophysiologies share several commonalities, which may aid in the treatment of chronic wounds to reduce inflammation and return the wound healing to its regular cycle.^26^

Common key processes end up becoming impaired as a result of a faulty wound-healing pathway. For example, Reich-Schupke *et al.* found that infections such as Methicillin-Resistant *Staphylococcus aureus* (MRSA) can become incredibly difficult to treat within a chronic wound, such as a leg ulcer, resulting in potentially life-threatening health risks such as sepsis and septic shock.^27^ Furthermore, certain underlying conditions, such as diabetes mellitus, can produce vascular complications that restrict angiogenesis and prevent the repair of damaged blood vessels, a crucial component of wound repair.^28^ Underlying health factors such as diabetes can also result in elevated levels of proteases such as matrix metalloproteinases (MMPs), which actively degrade the extracellular matrix (ECM), further increasing the risk for infection or poor wound healing outcomes.^29^ Examples of common chronic wounds and their associated conditions and presentations, as well as current treatment approaches, can be found below in Table 1.

**Table 1:** Common chronic wounds, their pathological features, conventional treatments, limitations, and the potential application of stimuli-responsive hydrogel strategies to address limitations

Chronic wound type	Pathological features	Conventional treatments	Limitations of conventional treatments	Stimuli-responsive hydrogel strategies
Diabetic Foot Ulcers (DFUs)^30,31^	Elevated matrix metalloproteinases (MMP) levels, chronic inflammation, impaired angiogenesis, and excess advanced glycation end-products (AGEs)	Debridement, offloading, infection control, and growth factor therapy	High recurrence rates, poor drug penetration	MMP or ROS-responsive hydrogels for controlled drug release, macrophage polarization agents, anti-glycation therapies
Venous Leg Ulcers (VLUs)^32,33^	Venous hypertension, fibrin cuff formation, inflammation, delayed epithelialization	Compression therapy, wound dressings, skin grafts	High recurrence rates, poor long-term healing	pH-Responsive hydrogels (targeting alkaline pH), ROS-responsive hydrogels, growth factor delivery
Pressure Ulcers (PUs)^34,35^	Ischemia, necrosis from prolonged pressure, bacterial colonization, biofilm formation	Offloading, debridement of devitalized tissue, drainage of infected areas	Difficult healing in advanced stages	Oxygen-releasing and ROS-responsive hydrogels, antibiofilm strategies

Several wound conditions, either as a result of existing medical conditions or characteristics of the wound's environment, can delay and disrupt the healing process, resulting in chronic wound formation. Among them, infection, oxygenation, and moisture levels have been shown to play a significant role in wound repair and could all potentially be addressed by artificial wound dressings capable of incorporating molecules that can target or recognize the needs of healing wounds.^18,36^ Edwards *et al.* proposed a pathway by which bacterial infection can disrupt the wound healing process due to excessive inflammation from an increase in cytokines that reduces the presence of growth factors in the wound site.^37^ Rodriguez *et al.* summarized the trends of oxygenation in chronic wounds, determining that the exact role and mechanism by which oxygen aids a wound environment must be investigated further, but patterns do appear to suggest that over- or under-oxygenation of chronic wounds delays and disrupts wound healing.^38^ Interestingly, they also found that high-stress lifestyles or medical conditions that decreased oxygen flow to chronic wound sites were correlated with poor wound healing outcomes, suggesting a connection between wound healing and other diseases or socioeconomic factors. Thus, it is imperative to investigate and design solutions that can address these areas of concern while maintaining adequate physical protection of the wound site and all other functionality expected of a wound dressing.

## 3 An overview of hydrogels

Hydrogels are three-dimensional (3D) networks of cross-linked water-soluble polymers that can retain large amounts of fluid and water without disrupting biomechanical integrity.^13^ Their high water retention capacity is attributed to hydrophilic functional groups such as hydroxyl (–OH), carboxyl (–COOH), and amine (–NH_2_) within the polymer network. Conventional (non-responsive) hydrogels have demonstrated considerable benefits in wound healing, including maintaining a moist wound environment, promoting autolytic debridement, and serving as vehicles for passive drug delivery. Advanced non-responsive hydrogel systems have also been engineered to incorporate features such as hemostatic effects, antimicrobial behavior, or bioactive additives for enhanced therapeutic outcomes.^39–41^

The amphiphilic properties of hydrogels makes them highly biocompatible with a wide range of molecules, making them a promising target for drug delivery systems. As a result of these favorable properties, both responsive and non-responsive hydrogels have increasingly become a focus point of research into chronic wound dressings in recent years.

Modern hydrogel innovations have improved their functionality as wound dressings through the incorporation of ROS-scavenging compounds,^42^ stimulus-responsive materials for controlled drug release,^43^ injectable drug carriers,^44^ conductive elements,^45^ hemostatic agents, and antimicrobial properties, among others. Compared to other wound dressings—such as gauze, foams, films, or hydrocolloids—hydrogels offer a unique set of advantages that are particularly relevant for chronic wound management.^46^ Their high water content not only maintains a moist wound environment critical for autolytic debridement and epithelial migration; it also provides inherent cooling and pain relief.^46^ The soft, conformable nature of hydrogels allows them to adapt to irregular wound geometries without damaging surrounding tissue. Moreover, their porous 3D network can serve as a scaffold for cell infiltration, angiogenesis, and extracellular matrix remodeling—key processes that are often stalled in chronic wounds.^47^ Hydrogels can also be loaded with bioactive compounds such as growth factors, antimicrobials, or stem cells, and their delivery can be spatially and temporally controlled in ways that are not feasible with more traditional materials. Beyond wound care, hydrogels demonstrated potential in other biomedical applications, such as 3D cell culture systems, targeted drug delivery, and tissue engineering, though these topics won't be explored in depth in this review.^48^ By extensively modifying the materials and conditions used in hydrogel formation, researchers continue to discover new ways to enhance their mechanical properties and performance.

## 4 Stimuli-responsive hydrogels

Stimuli-responsive hydrogels possess the ability to undergo structural and functional changes in response to specific environmental triggers. While non-responsive hydrogels already offer several therapeutic benefits, responsive hydrogels provide an added layer of adaptability, enabling real-time, on-demand reactions to changes in the chronic wound microenvironment. In chronic wound healing, where there is often an imbalance in one of these stimuli that contributes to delayed tissue repair, hydrogels can be tuned to release therapeutic agents in response to the wound's specific imbalance. The use of this stimulus-sensitive drug release enhances control over the healing process while also minimizing the need for frequent dressing replacement. This dynamic responsiveness makes stimuli-responsive hydrogels a more viable and adaptable alternative to conventional wound treatments. The following sections will explore the key types of stimuli that influence hydrogel behavior and their implications for the chronic wound healing process, including pH, temperature, and glucose levels.

### 4.1 Temperature-sensitive hydrogels

Temperature can be a strong indicator of the status of a wound, as it is influenced by important biological processes that aid tissue repair. During the first 3–5 days, wound site temperature rises in alignment with the inflammatory phase of normal wound healing.^49^ Moreover, an early sign of wound infection could be indicated by a significant increase of up to 2.77 °C in skin temperature, a characteristic that can be used to monitor chronic wound status and infection progression.^50^ This distinctive increase in temperature has the potential to be incorporated into controlled drug delivery systems in temperature-responsive hydrogels.

**Fig. 3:**
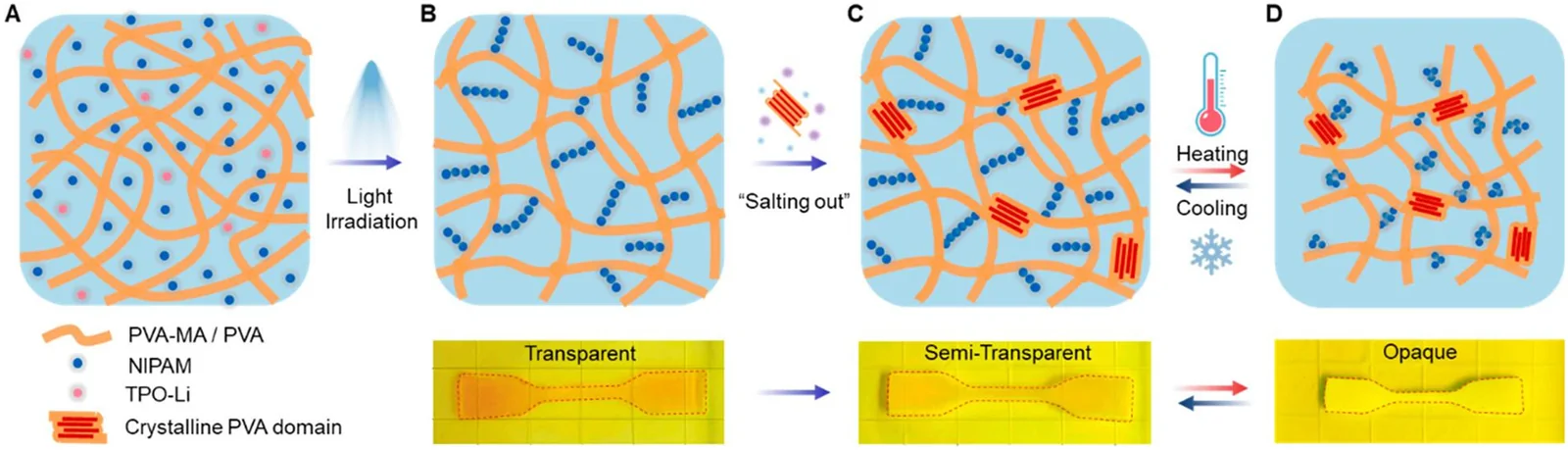
Illustration of heating and cooling of PVA/(PVA-MA)-*g*-PNIPAM hydrogel. (A) Aqueous precursor containing PVA, PVA-MA, NIPAM and TPO-Li. (B) One-pot synthesis of PVA/(PVA-MA)-*g*-PNIPAM hydrogel by light irradiation from a digital light processing 3D printer. The as-printed hydrogel was transparent. (C) Toughening of hydrogel by immersion in Na_2_SO_4_ salt solution to induce PVA aggregation and crystallization. The hydrogel turned semitransparent after the salting-out process. Upon heating, the hydrogel is actuated, turning into its coiled state represented in (D). The hydrogel turned completely opaque in this state after heating, and reverted back to its semitransparent state (C) after cooling. Reprinted and modified with permission from ref. 59, ACS, 2021.

Temperature-sensitive hydrogels undergo reversible phase transitions in response to temperature fluctuations. This thermoresponsive behavior is primarily due to the specific chemical composition and structure of the polymer chains, and is typically characterized by a specific lower critical solution temperature (LCST), at which the hydrogel's opaqueness, aqueous solubility, size, and shape are affected by temperature changes. Variations in LCST behavior can be due to the formation and dissociation of hydrogen bonds with water and interactions with polymer chain side groups, or it can be influenced by the balance between hydrophilic and hydrophobic interactions within the polymer.^51,52^ At a temperature below the LCST, the hydrogel is hydrophilic and in its swollen state. Once it reaches above this critical point, the hydrogel properties typically become hydrophobic and opaque, resulting in the expulsion of the content being contained in the mesh interpolymer networks. The LCST of the hydrogel can be modulated by incorporating various copolymers, changing the ratio of hydrophobic to hydrophilic components within the polymer matrix, or adding amino acids and different functional groups. Increasing the proportion of hydrophilic polymers such as when adding polyacrylic acid (PAA) (LCST = 56.3°) results in a corresponding elevation of the LCST, as the hydrophilic structures of the polymers have more interactions with water, specifically hydrogen bonds.^53,54^ Examples of synthetic polymers used in temperature-responsive hydrogels include poly(*N*-isopropylacrylamide) (PNIPAM), which has an LCST of 32 °C due to its hydrophobic isopropyl group and hydrophilic amide group;^55^ poly(vinyl alcohol) (PVA), whose LCST can be adjusted to the physiological temperature of 37 °C with just 10 mol% butanoylation;^56^ and poly(*N*-vinylcaprolactam) (PVCL), with an LCST ranging from 27 °C to 32 °C, influenced by its high molecular weight.^57^ Natural biopolymers such as chitosan and gelatin have also exhibited thermosensitive behavior.^58^Fig. 3 shows the morphology and image of what a typical thermosensitive hydrogel looks like above and below the LCST.

**Fig. 4:**
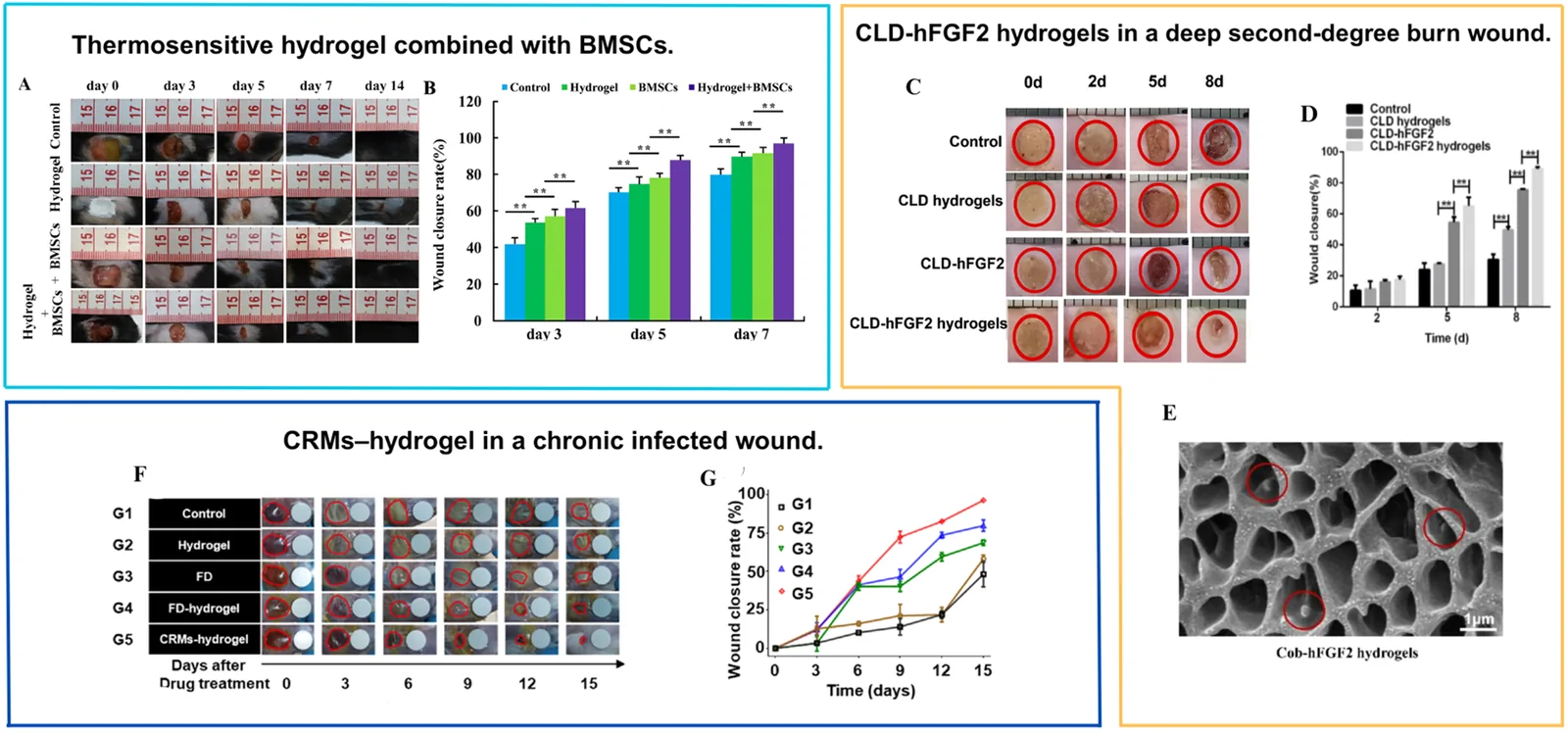
(A) General wound-healing conditions of the mice in the control group, the hydrogel group, the BMSCs group and the hydrogel-BMSCs combination group 3 days, 5 days, 7 days and 14 days after an operation creating a chronic wound. (B) Comparison of the wound-healing rates of mice in the control group, the hydrogel group, the BMSCs group and the hydrogel-BMSCs combination group 3 days, 5 days and 7 days after the operation. While the hydrogel and the BMSCs alone did display some improvement from the control, the combination of the two improved overall wound closure rates the fastest, suggesting that hydrogels can be more effective with combined aspects. (C) Images of deep second-degree burn wounds after being covered with PBS, CLD hydrogels, CLD-hFGF2, and CLD-hFGF2 hydrogels. (D) Wound healing rates on the 0th, 2nd, 5th, and 8th days (*n* = 6, ** *p* < 0.01). Closure percentages for CLD-hFGF2 and CLD-hFGF2 hydrogels were significantly higher than control for days 5 and 8. (E) Micromorphology of CLD-hFGF2 hydrogels observed using Cryo-SEM (red circle represents CLD-hFGF2). Displays the porous structure of hydrogel that allows successful release of the lipid droplets without damaging the structure after loading. The size of the CLD-hFGF2 is approximately 133.5 nm. (F) Representative chronic infected wound healing images on days 0, 3, 6, 9, 12, and 15 in control (G1), hydrogel (G2), mixtures of CIR and RIF (defined as FD) (curcumin, 2 mg per kg mice body weight; rifampicin, 1 mg per kg mice body weight) (G3), FD-hydrogel (G4), and CRMs-hydrogel (G5). (G) Time-wound closure rate in each group. Panels reproduced with permission from: A and B, ref. 61, Elsevier, 2018; C–E, ref. 62, MDPI, 2022; F and G, ref. 64, ACS, 2022.

By far the most commonly studied thermosensitive hydrogels are PNIPAM-based hydrogels, which are often copolymerized with additional hydrophobic or hydrophilic polymers to adjust the LCST, usually somewhere close to body temperature (36.7–37°).^60,61^ Due to their ability to prevent water and fluid loss from wounds and their adhesivity, thermosensitive PNIPAM hydrogels crosslinked with poly(amidoamine) (PAA) were used to encapsulate bone-marrow derived-mesenchymal stem cells (BMSCs) as a therapeutic approach to promote wound closure. As shown in Fig. 4A and B, this combination was shown to be biocompatible, significantly reduce the inflammatory response in wounds, and improve wound closure rates.^61^ The thermosensitivity of these hydrogels allowed them to achieve fast sol–gel transition once in contact with the wound, and their porous internal structure provided a good environment for cell proliferation, differentiation, and nutrient exchange. Additionally, thermosensitive poloxam hydrogels were developed by Zhang *et al.* to deliver human fibroblast growth factor 2 (hFGF2) linked with Camelina lipid droplets to accelerate burn wound healing in deep second-degree burns, which often lead to chronic wound complications.^62,63^ Poloxam blocks consist of hydrophilic ethylene oxide (EO) and hydrophobic propylene oxide (PO) blocks, with EO-POB-EOA as the basic structure. This structure allowed for hydrophobically assembled micelles to form in an aqueous solution in between chain segments, making it a proprietary drug carrier. The combination of the two also resulted in faster growth of new epidermis and wound closure rates, especially after 5 and 8 days (Fig. 4C–E). Zhao *et al.* used curcumin- and rifampicin-loaded micelles (CRMs) loaded in thermosensitive Pluronic F127 and F68 block polymers that were modified by the epidermal growth factor receptor (EGFR) targeted peptide GE11 and MMP9-responsive peptide PVGLIG.^64^ MMP9 is a matrix metalloproteinase found in high concentrations in a chronic wound environment. The thermosensitive hydrogel served as a highly adhesive base for the release of the CRMs, which demonstrated excellent intracellular and extracellular bacterial elimination efficiency, achieving an antibacterial rate of 99% in an MRSA-infected diabetic wound. While curcumin (Cur) and rifampicin (RIF) are hard to utilize in the body due to rapid metabolism, poor absorption as hydrophobic agents, and chemical instability, they are still favored for infected wound healing as they can promote cell migration and inhibit bacteria respectively, and using the thermosensitive hydrogel provides that ideal microenvironment for MMP9-responsive drugs to release in a chronic wound.^65^ As displayed in Fig. 4F and G, the drug-releasing system was found to effectively reduce inflammatory response and promoted the neovascularization and re-epithelialization process of chronic wounds in almost half the time it takes for the skin barrier to regenerate compared to the control.

In addition to facilitating drug release, temperature-responsive hydrogels can be designed to mechanically contract in response to body heat, thereby promoting physical wound closure.^66^ This is particularly advantageous in chronic wounds where wound edge retraction and impaired contraction delay healing. Certain thermosensitive hydrogels exhibit volumetric shrinkage above their LCST, generating mechanical tension that mimics myofibroblast-mediated contraction. For instance, a study by Chen *et al.* demonstrated that a core-ring PNIPAM-based shrinking hydrogel, when applied to chronic wound beds, produced directional traction forces that not only promoted tissue approximation but also accelerated re-epithelialization.^66^ Liu *et al.* also designed a wet-adhesive temperature-responsive hydrogel tape with a capacity for fast wound contraction under physiological temperature conditions.^67^ By aligning mechanical contraction with biological healing processes, these hydrogels offer a dual-function strategy—serving as both smart drug carriers and active mechanical agents that restore tissue integrity more efficiently.

### 4.2 pH-sensitive hydrogels

Normal, healthy skin typically maintains a slightly acidic pH range of 4 to 6.^68^ Chronic wounds, however, tend to have a higher pH, ranging from 7 to 9, due to the presence of blood, interstitial fluid, ammonia, and other factors.^69^ Additionally, if a wound becomes infected, its pH may decrease as bacteria produce acidic byproducts like lactic acid and carbonic acid.^70^ Thus, pH serves as a dependable indicator of wound status. The unique biochemical and mechanical characteristics of pH-responsive hydrogels, along with their ability to modify their morphology, volume, and medication release behavior, make them a valuable asset in wound care.^71^ These materials can effectively monitor wound status based on pH levels, facilitate controlled wound healing, mitigate infection risks, and expedite the healing process.^72^

Hydrogels exhibit different LCST ranges depending on the environmental pH values. Below their LCST, they exhibit small and broad pH sensitivities normally observed in most hydrophilic polyelectrolyte gels, but above their LCST, they exhibit sharp pH-dependent phase transition behaviors. The pH-dependent transition is strongly affected by temperature, while the temperature-dependent transition is, in turn, largely influenced by the pH.^73^ pH-Sensitive swelling occurs in ionic hydrogels due to the presence of charge-carrying pendant groups and is influenced by many factors, including ionic charge, p*K*_a_ or p*K*_b_ values of ionizable groups, degree of ionization, hydrophilicity, polymer concentration, and pH of the swelling medium. Among these factors, pH and the nature of pendant groups are the key factors for controlling the properties of pH-sensitive hydrogels. Cationic hydrogels swell at low pH due to protonation of amino/imine groups. These types of hydrogels can be used for antibiotic delivery to the stomach in cases of ulcerative conditions or as carriers for an injectable drug delivery system. Anionic hydrogels swell at higher pH due to ionization of the acidic groups. This property of hydrogels can be exploited for drug delivery at pH 7.4 in the intestine.^74^

**Fig. 5:**
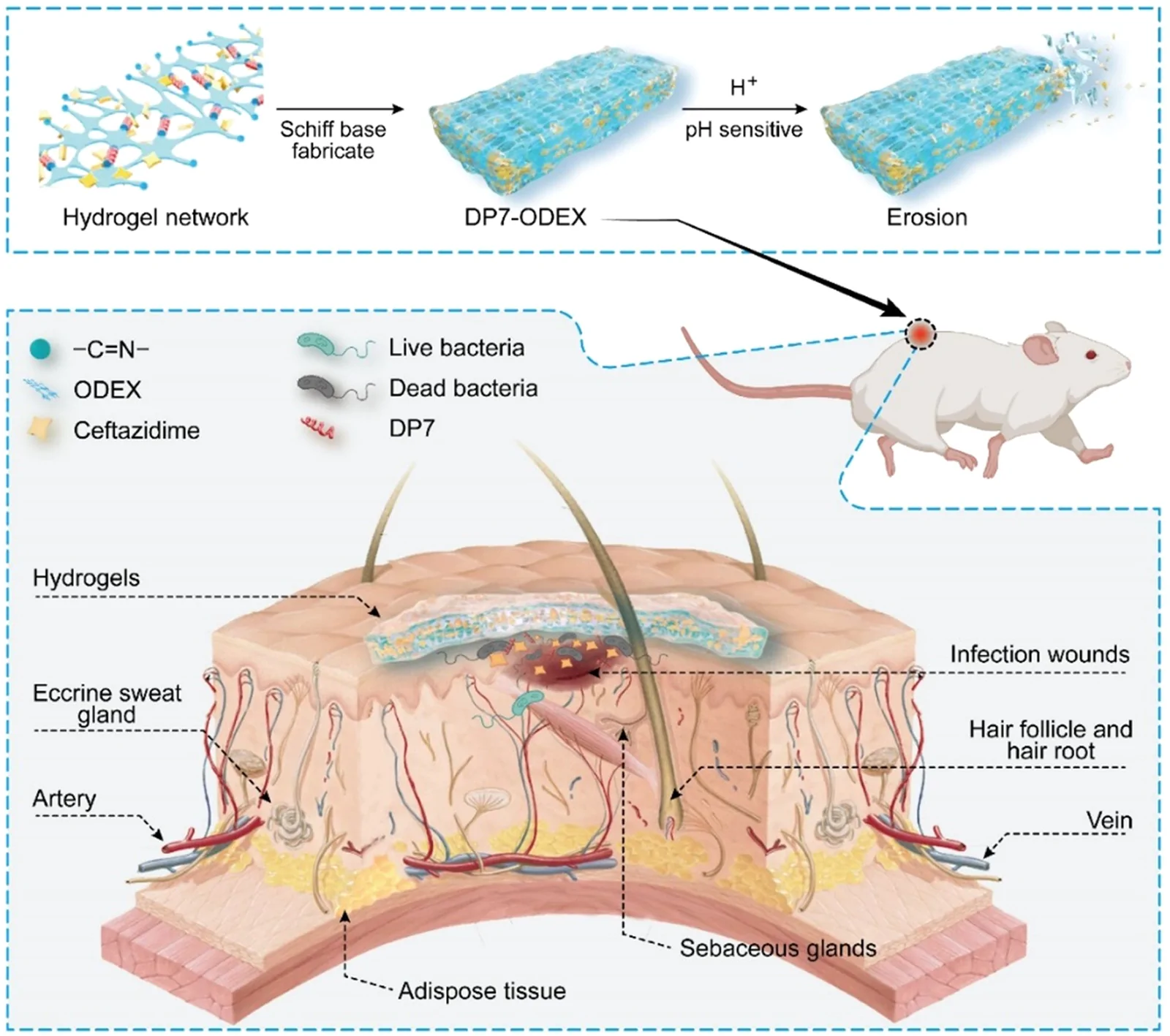
Structure of pH sensitive CAZ-DP7-ODEX hydrogel network and the mechanism for enhanced MDR bacteria infected wound healing process. This demonstrates how a pH-sensitive dextran-based hydrogel erodes and releases the drug in response to the acidic environment of an infected wound. Reprinted with permission from ref. 56, Elsevier, 2015.

Polysaccharide-composite hydrogels responsive to pH have been studied for their crucial role in wound healing. The most commonly used pH-sensitive hydrogels include chitosan, alginate, hyaluronic acid, guar-gum, and dextran-based gels.^75^ Infected areas in a wound often exhibit acidic pH levels, and polysaccharide dressings designed to respond to these conditions—either by releasing medication upon stimulation or by partially degrading the hydrogel to deliver the drug, as shown in Fig. 5—can enhance bactericidal activity, modulate inflammation, and promote better wound healing outcomes.^76^ The advantage of pH-responsive hydrogels over conventional hydrogels is the ability to release bioactive molecules—such as hemostatic agents (*e.g.*, thromboxane, gelatin, and chitosan), antimicrobial substances (*e.g.*, gentamicin, penicillin, and penciclovir), anti-inflammatory drugs (*e.g.*, vitamin E succinate, moxifloxacin, and curcumin), and epidermal growth factors (EGFs)—spatially and controllably at the wound locations, thereby accelerating the healing process.^77^

Polymers that show pH-responsive activity are a class of polyelectrolytes with ionic functional groups that are either weak basic or weak acidic groups.^78^ The vital element that leads to pH sensitivity in polymers is the presence of ionizable pendant groups that attach to the hydrophobic backbone of the polymer chain.^79^ The most commonly studied ionic polymers, their response range, and their benefits are listed in Table 2.

**Table 2:** Commonly studied pH-responsive polymers, their most effective pH response ranges, and benefits

pH-Responsive polymers	Most effective pH response range	Benefits
Poly(acrylic acid) (PAA)^80^	pH 6.0–7.7	Enables drug delivery to the specific cell domains of tissues with less acidic microenvironment. Can encourage the growth of granulation tissue, which aids in healing wounds and lays the groundwork for developing new skin.
Poly(acrylamide) (PAAm)^81,82^	pH 8.0–11.0	Has low toxicity and is economical. Has a large degree of swelling that enables the polymer to provide a moist environment in the wound area.
Poly(methacrylic acid) (PMAA)^83,84^	pH 5.8–6.1	Improve wound closure and vascularity *in vivo* by increasing the number of blood vessels of a wound in diabetic wounds.
Poly(diethylaminoethyl methacrylate) (PDEAEMA)^85^	pH 5.0–7.0	These polymers show low critical micelle concentration values which could markedly improve micelle stability and extend the range of applications in controlled drug delivery.
Poly(dimethylaminoethyl methacrylate) (PDMAEMA)^86,87^	pH > 7.4	Has excellent biocompatibility and can be quaternized for antibacterial activity, giving any hydrogels using this polymer a natural antibiotic, avoiding the use of drugs or potentially toxic metal nanoparticles.

### 4.3 Glucose-sensitive hydrogels

Glucose not only serves as a primary energy source for cells but also plays a crucial role in cell signaling pathways and immune response. Although if left unregulated, elevated glucose levels, otherwise known as hyperglycemia, can greatly hinder the healing process of wounds, impairing inflammation, proliferation, and remodeling during wound healing.^36^ Notably, elevated glucose levels cause constriction of the blood vessels and cell membrane rigidification, which limits oxygen and nutrient delivery to a wounded area.^88^ This reduced blood supply further slows the wound healing and can lead to tissue necrosis. Moreover, the elevated glucose levels disrupt the normal function of immune cells, hindering processes such as leukocytes’ abilities to swallow and kill harmful bacteria, which are essential for clearing wound infection and initiating tissue proliferation or repair. Along with the fact that high glucose levels can augment bacterial growth, this results in a prolonged healing process and higher infection rates of diabetic chronic wounds.^89^ Glucose-sensitive hydrogels can be designed as controlled-release drug delivery systems that respond to local glucose concentrations, either by leveraging the hydrogel's inherent properties or by incorporating functional groups that enhance glucose binding.^90^ These hydrogel membranes can be loaded with various medications, such as insulin, metformin, and l-arginine, to regulate glucose levels while also providing antibacterial protection.^91,92^ Common hydrogel polymers that are used with glucose-sensitive drug release systems include *N*-(3-d-glucose)acrylamide (GAA), polyacrylamide (PAM) based, polyvinyl alcohol (PVA), and chitosan-based hydrogels.^93^

**Fig. 6:**
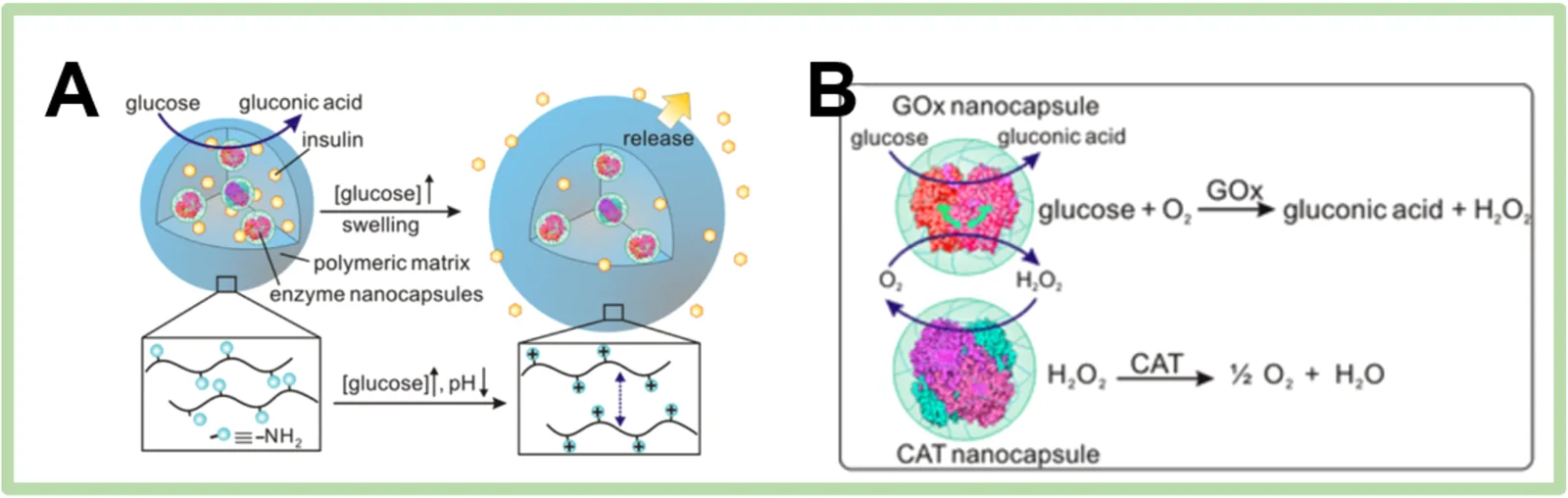
(A) Schematic of microgels encapsulating insulin and enzyme nanocapsules. The encapsulated glucose-specific enzyme catalyzes glucose into gluconic acid. The subsequent protonation of polymer chains with rich amine groups increases the charge in the gel matrix, leading to swelling of the microgels and release of insulin. (B) Enzymatic reactions involving glucose oxidase (GO_*x*_) and catalase (CAT) nanocapsules. Reprinted and modified with permission from ref. 97, ACS, 2013.

Glucose oxidase catalyzes the oxidation of glucose to gluconic acid using molecular oxygen (O_2_) as an electron acceptor, releasing hydrogen peroxide as a byproduct.^94^ Glucose-sensitive hydrogels based on a glucose oxidase (GO_*x*_) system typically use a pH-responsive hydrogel as the backbone, with GO_*x*_ immobilized into the matrix to impart glucose sensitivity. One common mechanism of a glucose-responsive hydrogel utilizes the pH fluctuation associated with GO_*x*_ catalysis of the conversion of glucose into gluconic acid and hydrogen peroxide (H_2_O_2_).^95^ Decreases in pH due to glucuronic acid and hydrogen peroxide production catalyzed by GO_*x*_ result in the pH-responsive hydrogel swelling and pore size increase, which in return triggers the release of loaded drugs.^96^ Gu *et al.* created injectable glucose-responsive microgels consisting of a physically cross-linked pH-responsive polymeric matrix, GO_*x*_- and catalase (CAT)-containing enzyme nanocapsules, and human recombinant insulin. CAT is used to regenerate oxygen, assisting in GO_*x*_'s glucose catalysis, and scavenge excess hydrogen peroxide produced in glucose oxidation.^97^ When glucose levels were elevated within the region, the GO_*x*_ and CAT reactions resulted in production of gluconic acid and hydrogen peroxide, which lead to a decrease in pH as previously described. The protonation of amino groups of the chitosan shell eventually triggered the swelling and dissociation of the hydrogel and resulted in the release of insulin.^97^Fig. 6 depicts a schematic of the hydrogel system and protonation of the amino groups that trigger insulin release, as well as of the enzymatic reactions that result in pH change.

**Fig. 7:**
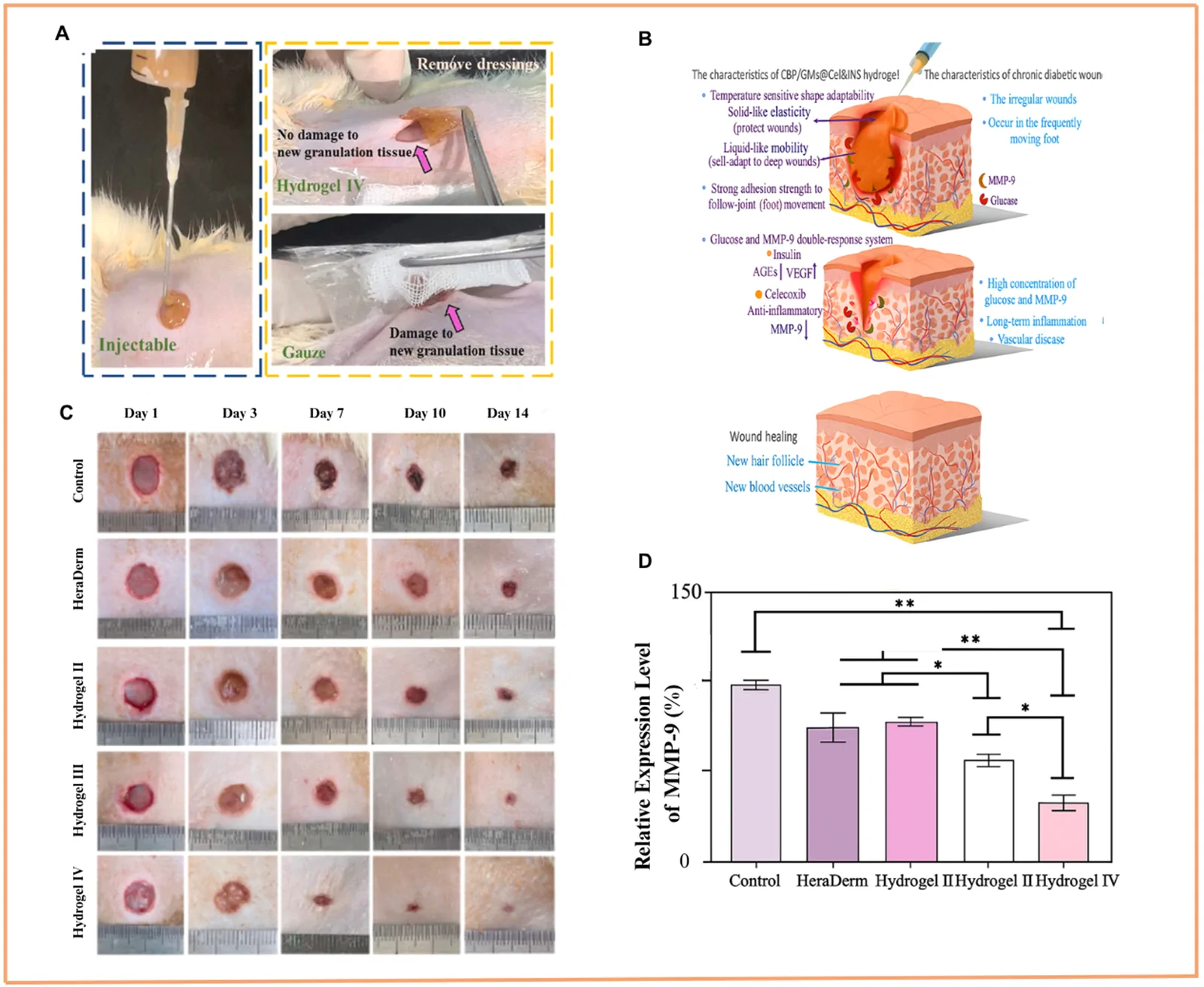
(A) Photographs of injection and stripping of hydrogels, and the adhesion of gauze to wounds during stripping. (B) Mechanism of wound healing induced by CBP/GMs@Cel&INS hydrogel. (C) Photos of the wounds at days 0, 3, 7, 10, and 14 hydrogel. (D) Immunofluorescence staining of MMP expression on wounds of different groups taken on day 7. Note: hydrogel II: the CBP/GMs hydrogel; hydrogel III: the CBP/GMs@INS hydrogel; hydrogel IV: the CBP/GMs@Cel&INS hydrogel. Reprinted and modified with permission from ref. 98, Elsevier, 2022.

Particularly, chitosan-based hydrogels with metformin have proven effective against diabetic foot wounds, which can pose an especially high risk of damage to other organs in patients as foot wounds especially receive constant trauma from repeated use.^43^ A study by Liang *et al.* using pH/glucose-responsive metformin-released hydrogels has proven quite effective in combating inhibited healing factors and symptoms associated with elevated glucose levels.^43^ By combining the hydrogel system with dihydrocaffeic acid (DA) and l-arginine, this resulted in improved tissue attachment, promoted healing, and increased vascularization. DA was added to improve antioxidant activity and decrease chronic inflammation, while l-arginine cografted with chitosan had a cationic effect that killed bacteria and an improved tissue adhesion between the wound and hydrogel dressing. This provides the hydrogel with effective antibacterial properties, helping the body overcome the compromised immune activity within the region as it was able to thoroughly inhibit *E. coli* and MRSA bacteria.^43^ As this system is responsive to both pH and glucose, the dressing exhibited increased metformin release under acidic/lower pH conditions and elevated glucose levels independently. The 1,2 diol structure in glucose can compete with the complex of catechol and phenyl boronic acid that encapsulates the metformin within the hydrogel, allowing the hydrogel network to dissociate and release metformin.^43^ Thus, increasing/higher glucose level is associated with increased metformin delivery.^43^ From a different perspective, Zhou *et al.* designed a glucose and matrix metalloproteinase-9 (MMP-9) responsive temperature-sensitive shape self-adaptive hydrogel (CBP/GMs@Cel&INS) using polyvinyl alcohol (PVA) and chitosan grafted with phenylboronic acid (CS-BA).^98^ These gels contained an anti-inflammatory drug, celecoxib, encapsulated in insulin and gelatin microspheres (GMs@Cel) that were released on demand under wound environments consisting of high concentrations of glucose and MMP-9, mimicking chronic diabetic wounds. This dressing had good adhesive and mechanical properties, which improved the drug release efficiency of insulin and celecoxib microspheres and reduced risk of infections, promoting excellent biocompatibility, cell proliferation, and migration. Ultimately, this resulted in the fastest rate of wound healing while promoting cell proliferation, migration, and glucose consumption. There was also found to be down-regulation of the expression of downstream MMP9, which has significant positive effects on chronic diabetic wounds,^90^ shown in Fig. 7D along with the mechanism, injection process, and wound healing results of this study (Fig. 7A–C).^98^

Glucose-sensitive hydrogels can also use phenylboronic groups (*e.g.* poly3(acrylamido)phenyl boronic acid) and polyol polymer (*e.g.* PVA) to form a gel substance with complex formation between the phenyl borate and hydroxyl groups.^99^ When these complexes are introduced to glucose, the pendant hydroxyl groups of glucose compete with the pendant phenyl borate and hydroxyl complexes of the original hydrogel, causing breakages in the crosslinked hydrogel. As the crosslinking density within the gel decreases in response to increasing glucose concentration, the gel swells and releases drugs. Elsherif *et al.* found that phenylboronic acid (PBA) is favored in the development of glucose-sensitive sensors because of its strong affinity for glucose under physiological conditions, simultaneous insulin release during glucose detection, non-toxicity, responsiveness to pH and glucose level changes, its ability to modify its chemical properties with different substituents, and high stability.^100^ Using light transmission, researchers tested the hydrogel's permeability when introduced to glucose. Consistent with expectations, increased glucose exposure resulted in increased maximum transmitted light power. Furthermore, they found that increased pH was correlated with increased glucose sensitivity, with the highest sensitivity at a pH of 8. Both the ionic strength/pH and the glucose concentration of the solution greatly affect this phenylboronic acid hydrogel's ability to work effectively.^100^

Similar to phenylboronic acid-based hydrogels, free glucose can competitively bind to lectin in hydrogel networks and reduce crosslinking, affecting membrane swelling and molecular release. This property has been widely explored for insulin use, as well as glucose-sensitive biosensors and wound healing applications. Notably, concanavalin A (Con A), a plant lectin purified from jack beans, has been widely used to modulate drug delivery, as it binds with a high affinity to glucose.^99^ A study by Hu *et al.* developed a Con A-based hydrogel fixed with green-synthetic silver particles on laser direct-writing graphene electrodes as a flexible continuous glucose monitoring (CGM) system.^101^These technologies are attractive as they monitor glucose levels at a low cost with good stability and high sensitivity. In this case, green-synthesized silver particles are a flexible enzyme-free glucose sensor prepared on laser-written graphene. Compared to Go_*x*_, this non-enzymatic system may have the ability to detect a larger range of glucose levels as opposed to enzymatic biosensor systems. Con A compaction was noted to increase crosslinking, reduce water absorption, minimize swelling, and slow drug release in aqueous environments, showing promising usage with non-enzymatic sensors. Additionally, the Con. A-based hydrogel sensors were found to have improved biocompatibility and stronger specificity to glucose compared to phenylboronic acid-based gels, suggesting potential for broader applications in glucose-responsive drug delivery, biosensing, and wound healing beyond insulin modulation. The competitive binding of DexG-Con A and glucose (Glu)-Con A influences the structure of the DexG-Con A network, leading to alterations in the hydrogel's volume and permittivity, resulting in the release of insulin.^101^ Con A molecule preferentially binds with glucose rather than DexG, thus the presence of glucose in the DexG-Con A system affects the permeability of the membrane due to disruption in crosslinking of DexG-Con A. These hydrogels reflect another promising method of glucose regulation, wound healing, biosensing, and drug delivery due to their strong specificity to glucose in the bloodstream.

Furthermore, glucose-sensitive hydrogels have also been explored for controlled release of antimicrobial agents in wound healing applications, where elevated glucose levels in diabetic wounds can trigger the localized release of antimicrobial components.^102^ These hydrogels, in response to hyperglycemia, can not only release agents from the hydrogel that consume glucose in the bloodstream, stabilizing the environment, but can also simultaneously deliver antimicrobial agents to handle any hazardous microbes that could have formed and thrived in the hyperglycemic environment.

### 4.4 ROS-sensitive hydrogels

ROS play a dual role in wound healing, being essential for defense against pathogens but harmful when present in excess. ROS are byproducts of cellular metabolism that consist of partially reduced metabolites of O_2_, which are a necessary factor in yielding mitochondria-driven ATP. Without these cellular processes, wounds would not have the necessary energy to heal. They serve as important secondary messengers regulating physiological functions of macrophages, and are also known to mediate angiogenesis and vasoconstriction/vasodilation at wound sites. On the other hand, excessive ROS production levels can cause oxidative stress on tissues and cellular damage, slowing the process of wound repair. Chronic wounds have historically been characterized by prolonged inflammation and elevated ROS levels, which are known to cause damage to tissues and other important healing factors.^103^ This factor makes ROS a suitable trigger stimulus for hydrogel designed to improve healing and inflammation in chronic wounds.

**Fig. 8:**
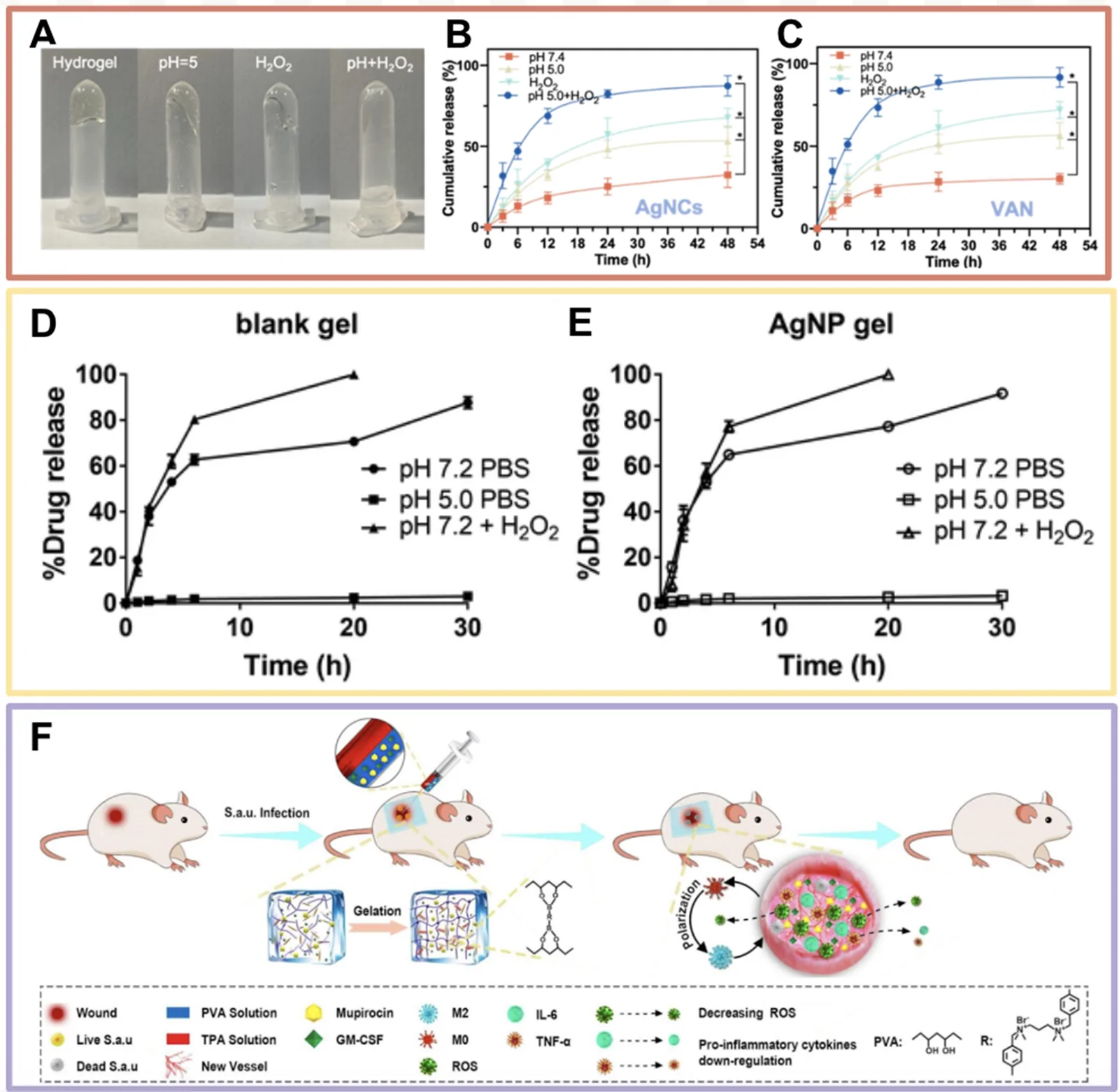
(A) Images of the hydrogel after being incubated with PBS (pH = 5) and/or H_2_O_2_. Incubation in the H_2_O_2_ solution induced hydrogel collapse and led to a liquid resembling state of the gel, as well as in the acidic PBS solution. The combination of both factors dissolved the hydrogel more comprehensively. (B) Release curve and characteristics of blank conjugated silver nanoclusters (AgNC) in 48 hours, which serve as encapsulated carriers for the antibacterial drug (mean ± SD, *n* = 3), *P < 0.01. (C) Release curve and characteristics of antibacterial agent vancomycin (VAN) in 48 h (mean ± SD, *n* = 3), *P < 0.01. (D and E) Graph demonstrating pH and ROS responsive drug release properties in a (D) blank and (E) AgNP hyaluronic acid–phenylboronic acid–tannic acid hydrogel. Buffer pH 7.2 with 1 mM H_2_O_2_ conditions were used to mimic chronic wound conditions while pH 5.0 PBS represented healthy skin pH. Fluorescein isothiocyanate (FITC) labeled bovine serum albumin (BSA) was used as a model drug. FITC-BSA showed the fastest release in PBS at pH 7.2 with 1 mM H_2_O_2_, a moderate release in PBS at pH 7.2, and a very slow release in PBS at pH 5.0 for both gels. (F) Schematic illustration of using the ROS-responsive hydrogel loaded with therapeutics for treatment of wounds with bacterial infection. Panels reproduced with permission from: A–C, ref. 104, ACS, 2021; D and E, ref. 105, RCS, 2021; F, ref. 106, Elsevier, 2020.

Hydrogels can be engineered with ROS-sensitive linkers or components that degrade or undergo structural changes when exposed to high concentrations of ROS, consequently triggering the release of therapeutic agents. These reactions occur when ROS, such as hydrogen peroxide (H_2_O_2_), superoxide (O_2_^−^), or hydroxyl radicals (˙OH), oxidize or break down certain functional groups or bonds in the hydrogel. For instance, in the presence of a solution of 1 mM H_2_O_2_, a phenylboronic acid–diol ester bond was shown to dissolve a phenylboronic acid modified hydrogel, giving it the capability to release antibacterial carriers and drugs on demand, as shown in Fig. 8A–C.^104^ Another example includes an injectable loaded hydrogel with tannic acid loaded silver nanoparticles, placed in an environment mimicking chronic wound conditions.^105^ In the presence of ROS, the rate of the drug release and hydrogel degradation both increased compared to an environment without ROS present. This increased rate was determined to be caused from the degradation of a boronic ester together with the oxidation of TA into quinone, leading to the entirety of the drug being released within 20 hours, as shown in Fig. 8D and E.^105^ Moreover, Zhao *et al.* used a ROS-responsive linker in a PVA based hydrogel to release an antibiotic (mupirocin) and growth factor (GM-CSF) upon contact with ROS in both healthy and diabetic mice, with a schematic displayed in Fig. 8F.^106^ As the ROS-linker (*N*1-(4-boronobenzyl)-*N*3-(4-boronophenyl)-*N*1,*N*1,*N*3,*N*3-tetramethylpropane-1,3-diaminium (TPA)) scavenged the oxidative environment, pro-inflammatory cytokines were simultaneously downregulated and M2 phenotype macrophages were up-regulated, resulting in increased angiogenesis, revascularization, and production of collagen. At the same time, the hydrogel gradually degraded as a result of the cleavage of the linkers, and released the encapsulated antibiotics and growth factors, eliminating *Staphylococcus aureus* (S.A.U.) infection and accelerating wound repair.^106^

### 4.5 Matrix-metalloproteinase-sensitive hydrogels

The matrix-metalloproteinase (MMP) family is a group of calcium-dependent zinc-containing enzymes that are involved in the degradation of various ECM components. MMPs are present in both acute and chronic wounds, regulating ECM degradation and deposition, which is essential for wound reepithelialization. The excess protease activity can prevent wound closure and increase the risk of bacterial infection, leading to a chronic nonhealing wound.^107^

MMP-responsive hydrogels are particularly suited for chronic diabetic wounds because these wounds consistently exhibit elevated levels of MMPs, especially MMP-9 and MMP-2. The overexpression of MMP-9 in wounds is a significant contributing factor to excessive ECM degradation, which hinders the process of wound healing.^108^ In 2025, Lin *et al.* designed an MMP-9 responsive injectable duplex hybrid hydrogel with oxidized hyaluronic acid and hydrazide-grafted gelatin, which are known to degrade in the presence of MMP-9.^109^ The hydrogel was mixed with the drug cinnamaldehyde (CA) and crosslinked into a gel after local *in situ* injection into the wound site. Then, under the action of MMP-9, the gelatin degraded and continuously released CA, inhibiting cell death of traumatic endothelial cells and accelerating healing of diabetic wounds.^109^

In summary, MMP-responsive hydrogels offer a targeted strategy for chronic wound management by leveraging elevated protease activity to enable controlled degradation and localized therapeutic release. This responsive behavior helps restore ECM balance, reduce inflammation, and accelerate tissue regeneration, making them especially promising for treating chronic diabetic wounds.

### 4.6 Mechanoresponsive hydrogels

Hydrogels, as a result of their flexible and adhesive characteristics, have recently been investigated as a potential therapeutic option for wound sites subject to high mechanical forces. Examples of these sites include the elbows, wrists, ankles, and knees, where typical wound dressings may struggle to stay in place and maintain an ideal wound environment when subject to mechanical loads.^110^ Yu *et al.* compiled the mechanical properties of several hydrogel compositions with a combination of both physical and chemical cross-linking elements.^110^ They were able to find hydrogel designs optimal for high mechanical loads at these movable sites, such as the GT/EDC–NHS–DA nanofibrous hydrogel designed by Zheng *et al.* which boosted tensile strength by over 200% compared to a non-dopamine-grafted GT gel, and the QCS/PF hydrogel synthesized by Qu *et al.* which exhibited highly reversible elongation and joint flexural properties similar to that of natural skin.^111,112^ However, while these hydrogels boast impressive mechanical properties and offer a solution for complicated wound sites in high-load regions of the body, the high degree of chemical cross-linking often comes at the compromise of other bioactive capabilities such as antibacterial activity.^110^

While mechanical forces have always been a consideration in designing and testing hydrogel wound dressings to closely match the properties of the skin or tissue where they might be used, they also play a role as a potential stimulus to encourage a change in the physical and chemical properties of a mechanoresponsive hydrogel.^113^ For example, hydrogels designed to simulate soft tissues can exhibit strain-stiffening when subjected to an applied stress, a property exhibited naturally by these tissues. Song *et al.* outlined the strain-stiffening mechanism through the addition of polymers such as actin-binding proteins (ABPs) and demonstrated that, while not a perfect match, this behavior closely mimics the natural mechanics of soft tissue in the body.^114^ Fang *et al.* demonstrated the potential therapeutic power of strain-stiffening by developing a mechanoresponsive poly(sulfobetaine methacrylate) hydrogel, which delivered a loaded antibacterial drug into the wound site following application of mechanical force.^115^ This hydrogel maintains the flexibility and biocompatibility of typical cross-linked hydrogels with the added bonus of a controlled drug-release system based on the application of stress through stretching or compression of the wound dressing. Mehnath *et al.* prepared a similar mechanoresponsive hydrogel specifically aimed at diabetic foot ulcers and other chronic wounds, and were able to show that application of a mechanical force was able to produce greater drug delivery to the wound site.^116^ The role of mechanical force as both a metric of hydrogel stability on difficult wound sites and as a stimulus to trigger drug release will likely continue to be investigated, given the success of existing efforts, and should be closely observed as a method to improve outcomes for individuals with chronic wounds.

### 4.7 Magnetic-responsive hydrogels

Magnetic nanoparticles (MNPs), which typically range from 1 to 100 nanometers in size, are characterized by the small particle size, larger surface properties, magnetic response, and superparamagnetism. Notably, they have been recognized as a promising component in enhancing the wound healing capabilities of the standard hydrogel. Iron oxides, Fe_3_O_4_ and Fe_2_O_4_, are commonly modified into nanocomposites for usage in hydrogels because of their good chemical stability, high magnetization, and biocompatibility.^117^ By incorporating MNPs into hydrogel matrices, researchers have developed smart biomaterials that can be externally controlled and tailored to support the wound healing process.

In the context of chronic wound healing, magnetic-responsive hydrogels offer significant advantages. Chronic wounds often stall in the inflammatory phase and suffer from poor vascularization, persistent infection, and impaired tissue regeneration. Hydrogels with MNPs can combat these complications due to their unique capabilities. When an external magnetic field is applied, the embedded MNPs create micro-level mechanical forces within the hydrogel matrix; these forces can be sensed by surrounding cells that promote cell adhesion, spreading, and division.^118^ Furthermore, when an alternating magnetic field is applied, MNPs within the hydrogel absorb electromagnetic energy and convert it into heat. This localized heat can be sufficient in killing the bacteria without damaging the surrounding healthy tissue.^119^ This heating can also enhance the effectiveness of drugs delivered at the site. The grafting method in MNP synthesis requires that the magnetic particles and the hydrogel monomers are covalently bound together with the assistance of micro or nano crosslinkers grafted to the magnetic particles. This method results in the most stable hydrogel networks, as there are no bonding interactions between MNPs and the hydrogel networks of hydrogel products of the *in situ* and blending methods.^120^

Many recent studies have explored application of magnetic-responsive hydrogels to accelerate wound healing. In one study, Ye *et al*. developed chitosan polyvinyl alcohol hydrogel beads (MCPHBs). By incorporating iron II and III into hydrogels and forming MNPs through *in situ* using ammonium hydroxide, researchers created a product capable of responding to magnetic fields.^121^ These hydrogels demonstrated a great drug loading capactiy, making them suitable for long-term wound applications. In another study, He *et al.* engineered a multifunctional hydrogel dressing coated with protein fibers and embedded with antibacterial magnetic nanoactuators.^122^ Under external magnetic stimulation, these nanoactuators generated localized mechanical cues/signaling that activated skin cells, enhanced fibroblast migration, and promoted a more balanced healing response. Furthermore, with increased near-infrared irradiation stimulation, the hydrogel possessed better antimicrobial effect against *E. coli* and *S. aureus*. This dressing was able to regulate both pro-inflammatory and pro-regenerative signals, contributing to improved tissue regeneration without the complications of excessive scarring or fibrosis. These studies highlight how the abilities (capabilities, properties) of modified NMPs hydrogels can be harnessed for more precise control over the wound healing process.

### 4.8 Ultrasound-responsive hydrogels

Ultrasound-responsive hydrogels represent an emerging class of smart wound dressings that utilize non-invasive acoustic stimuli to modulate drug release, enhance tissue regeneration, and facilitate real-time treatment adaptation. Chronic wounds, particularly those associated with diabetes or infection, often require prolonged and precisely timed delivery of therapeutics to overcome barriers such as biofilm formation, inflammation, and impaired vascularization. Ultrasound, often referred to as sonophoresis, is seen as an attractive external stimulus as it can be controlled remotely, spatially, and temporally.^123^ Also, its ability to penetrate deep tissue and act on cells either thermally or mechanically gives it clinically relevant benefits.^124^ Using heat, cavitation, and acoustic flow, ultrasound-responsive hydrogels typically contain nanospheres that have components that respond to ultrasound to release therapeutic agents on demand.^123^

**Fig. 9:**
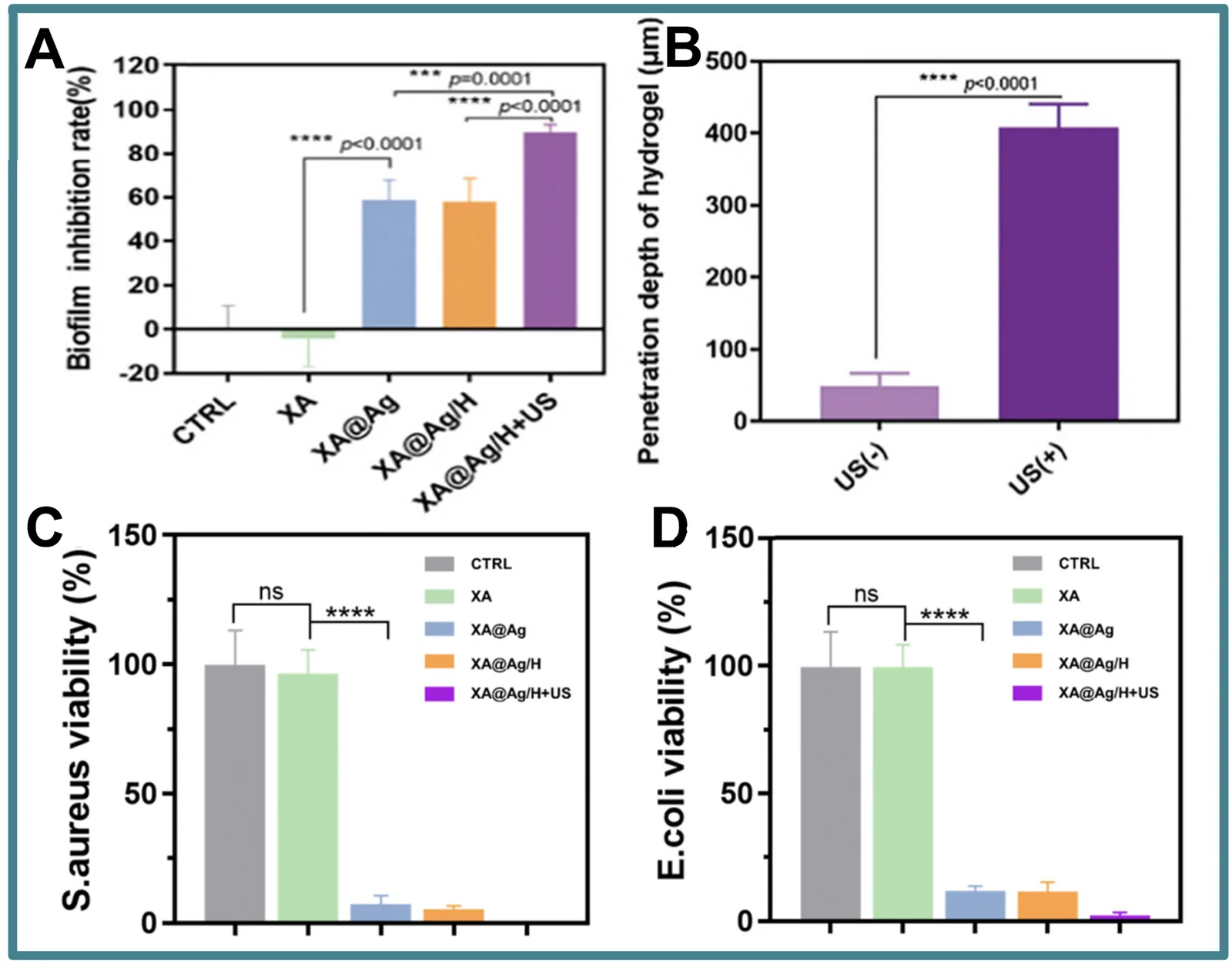
(A) Inhibition rate of *S. aureus* biofilm after treatment with different hydrogels, including a control, xanthan gum/sodium alginate composite hydrogel, composite hydrogel with encapsulated silver ions, 3D dual-network skeleton structure hydrogel with cross linking initiated by the addition of Ca^2+^ ions, and a 3D dual-netwok hydrogel with ultrasound. (B) Delivery depth of ultrasonic responsive hydrogel in skin wound, with and without ultrasound activation. (C and D) Relative bacterial viability of *S. aureus* and *E. coli* calculated based on the number of colonies on agar plates. Reprinted with permission from ref. 125, Wiley Advanced, 2024.

Ultrasound is commonly combined with a hydrogel drug delivery system as the soft, hydrated nature of hydrogels transmits ultrasound energy efficiently, enabling responsive elements within the matrix to react in a controlled manner. In addition, hydrogels have the capacity to localize therapeutic agents at the wound site, protect sensitive biomolecules from degradation, and maintain a moist, biocompatible environment that supports healing. Recent advances have demonstrated how ultrasound can be used not only to control drug release but also to facilitate biofilm disruption and improve therapeutic penetration in chronic wounds. For example, Zong *et al.* developed an ultrasound-responsive hydrogel embedded with self-assembled heparin-binding domain (HBD) peptide nanoparticles, designed specifically for diabetic wound healing complicated by biofilm infection.^125^ Biofilms are communities of bacteria encased in a protective extracellular matrix that commonly form on chronic wound beds, and they impede healing by promoting persistent inflammation, evading immune responses, and significantly reducing the efficacy of antibiotics. Upon ultrasound stimulation, the hydrogel underwent structural collapse, enabling controlled release of the embedded peptides and mechanical disruption of the biofilm matrix. This dual-action system enhanced tissue penetration while displaying significant antibacterial and antibiofilm activity, as shown below in Fig. 9.

Additionally, Huang *et al.* developed an injectable PEG-based hydrogel integrating food-derived anthocyanidin as a visible pH sensor and PLA microcapsules for ultrasound-triggered antibiotic release.^126^ The system allows real-time visual monitoring of wound pH, enabling early detection of infection. Upon ultrasound application, the microcapsules release antibiotics on demand, significantly reducing bacterial load in infected and diabetic wound models. This combined sensing-treatment strategy accelerated healing—confirmed by histology and reduced inflammatory cytokines—demonstrating a powerful example of smart, responsive wound dressings.

## 5 Additional functionalities

With the advancement of stimuli-sensitive components in hydrogel dressings, recent developments in stimuli-responsive hydrogels have introduced additional functionalities to further enhance chronic wound healing. ROS scavenging capabilities help mitigate oxidative stress, thereby reducing inflammation and promoting tissue regeneration. Meanwhile, hemostatic properties support rapid blood clotting, minimizing excessive bleeding and creating a stable environment for healing. This section explores how these integrated functionalities work synergistically with novel hydrogel dressings to improve wound recovery, offering a more effective approach to addressing the complex challenges of chronic wounds.

### 5.1 ROS antioxidant scavenging

In addition to ROS-sensitive drug release, ROS-scavenging functionality has been incorporated into hydrogels, which use antioxidants to modulate ROS levels at wound sites, fostering a supportive environment for tissue repair. During the inflammation stage, excess ROS are produced, which may inhibit the continuation of proper wound healing.^127^ Antioxidants are proteins that play a crucial role in helping maintain ROS homeostasis in the body by donating electrons and stabilizing excess ROS. This neutralizes their potential to damage critical cellular structures like lipids, proteins, and nucleic acids. Examples of ROS-scavenging hydrogel loading materials may include polyphenols such as curcumin, tannic acid, and dopamine, known for their exemplary biocompatibility and immunomodulatory activities.^42,128^ For instance, incorporating polydopamine-modified fullerene nanocomposites (C60@PDA) has demonstrated favorable cytocompatibility, hemocompatibility, and antibacterial properties in a full-thickness wound mouse model.^127^ Moreover, it has been shown to enhance re-epithelialization, collagen deposition, and angiogenesis. Fullerene, a potent antioxidant, possesses anti-inflammatory, antiviral, antibacterial, and hair growth-stimulating properties and has been previously utilized in whitening, anti-aging, and sunscreen cosmetic applications. However, due to its insolubility in aqueous solutions, fullerene was coated with polydopamine to enhance its bioavailability, tissue adhesiveness, and antibacterial efficacy.^127^

**Fig. 10:**
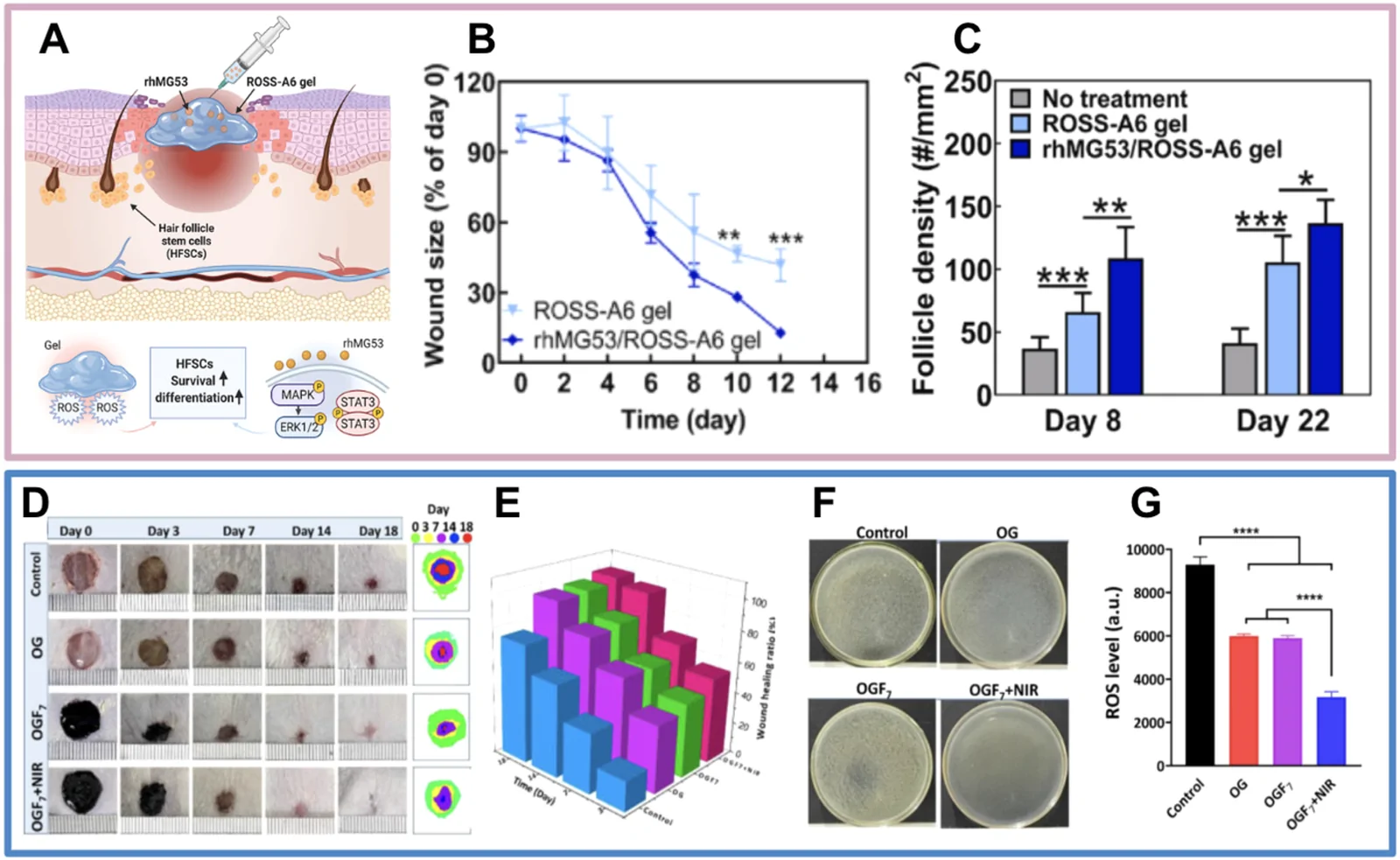
(A) Schematic illustration of accelerated wound healing by the delivery system of rhMG53/ROSS-A6 gel. The ROS scavenging hydrogel allowed the released human recombinant MG53 (hrMG53), a tripartite motif family protein, to proliferate longer under oxidative stress, thereby improving HFSC survival and enhancing wound healing. (B) Wound closure for 12 days for each treatment category. Wound size ratios were normalized to day 0. Data were expressed as means ± standard error (*n* ≥ 6, **p < 0.01, ***p < 0.001). (C) Quantification of hair follicle density *in vivo* based on cytokeratin 14 (K14) staining images (10× and 40×) illustrated to significantly promote HFSCs density at both day 8 and day 22 in the gel group compared with no treatment group (*n* = 8, *p < 0.05, **p < 0.01, ***p < 0.001). (D) Evaluation of OGF7 hydrogel on healing of *S. aureus* infected skin wound in STZ-induced diabetic mice. Photographs and wound contraction diagram of healing wounds on day 0, 3, 7, 14 and 18 in each group; (E) wound healing ratio in each group; (F) *Staphylococcus aureus* detection for analysis of wound infection on day 1; (G) ROS detection for analysis of oxidative stress on day 7. Panels reproduced with permission from: A–C, ref. 129, Elsevier, 2022; D–G, ref. 130, Elsevier, 2023.

Additionally, a study by Niu *et al.* used a ROS-scavenging hydrogel made of NIPAAm, HEMA and acrylated HPPE (AHPPE) (abbreviated as ROSS-A6) to promote hair follicle development and improve diabetic wound closure (Fig. 10A–C).^129^ Using AHPPE to scavenge and reduce ROS levels, the hydrogel was able to gradually release recombinant human MG53 (rhMG53), a key protein involved in cell membrane repair, to boost tissue regeneration and promote the survival of hair follicle stem cells (HFSC) under oxidative stress.^129^ The rhMG53 was expected to continuously release from the hydrogel after 21 days until all of the protein was released, which is especially useful as it can reduce the need for frequent drug administration, and is thus beneficial for chronic conditions. He *et al.* had also developed a hydrogel utilizing antioxidants to reduce ROS concentrations in infected diabetic mice wounds using a combination of oxidized dextran, gallic acid-grafted gelatin, and ferric ion (abbreviated as OGF).^130^ This hydrogel also incorporated a photothermal antibacterial aspect, using near infrared light (NIR) to denature bacterial proteins with heat energy. When combined with the successful mitigation of oxidative stress, this resulted in the removal of infection and full re-epithelialization of a *Staphylococcus aureus*-infected wound in diabetic mice within 18 days (Fig. 10D–G).^130^

### 5.2 Hemostatic effects

In emergency medicine and hospital care, hydrogels paired with hemostatic agents are gaining traction due to their superior ability to control severe external bleeding.^131^ Specifically, as a result of the unpredictable and dynamic environment in emergency and prehospital care, hydrogels that can function on humid or dirty skin with proper adhesion to encourage hemostasis are becoming a focus of hydrogel research for wound care. In hopes of enhancing adhesion in these challenging settings, researchers are exploring bioadhesive polymers, surface modifications, and fast-gelling formulations that allow hydrogels to adhere effectively despite the presence of moisture or contaminants. Some hydrogels incorporate catechol-based chemistry, inspired by mussel adhesion, or utilize stimuli-responsive crosslinking to rapidly bond with tissue, ensuring quicker hemostasis even in high-fluid environments.^132^

**Fig. 11:**
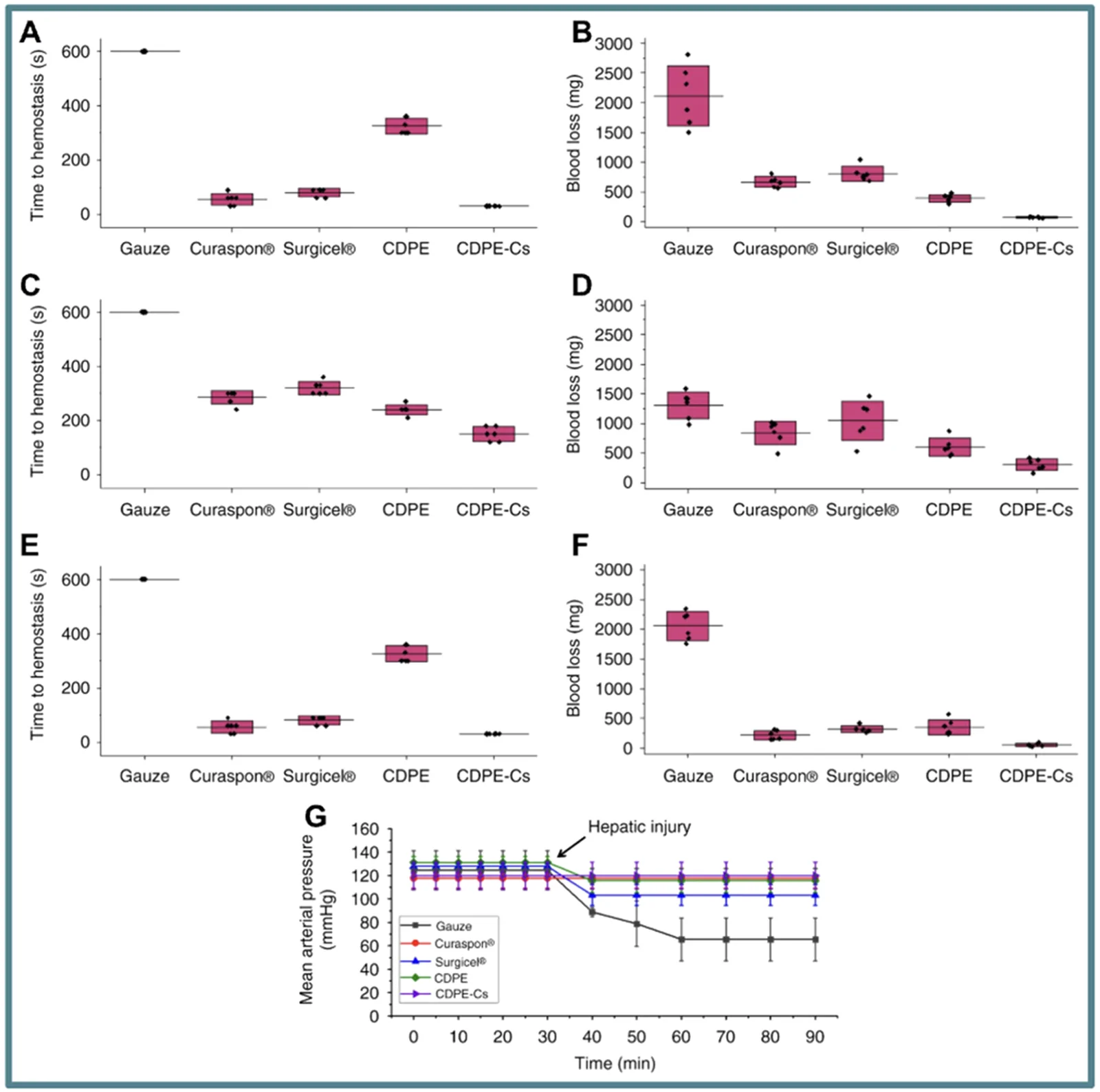
(A) Box plot of time to hemostasis in rat subjects following a liver laceration and the application of several commercially available hemostatic options as well as the β-cyclodextrin polyester (CDPE) and chitosan-loaded cyclodextrin (CDPE-Cs) hydrogels. (B) Graph showing blood loss from the wound site for each hemostatic option. (C)/(D) The results of a repeat of the experiment in rabbits, and similarly, (E)/(F) in pigs. (G) Mean arterial pressure *versus* time following the liver laceration in rabbits for several hemostatic options and the CDPE and CDPE-Cs hydrogels. Reprinted with permission from ref. 135, Nature, 2019.

During hemostasis, hydrogels can improve the wound healing process by promoting physical adhesion of underlying tissue or by providing the wound site with coagulation cofactors that aid in chemical wound healing processes. Ma *et al.* established a hydrogel cross-linked with a polymer containing chitosan and *o*-nitrobenzyl alcohol (termed the NB-CMC hydrogel) which demonstrated enhanced hemostatic effects and improved wound healing *in vivo.*^133^ Song *et al.* also utilized similar building block polymers such as *o*-nitrobenzyl in an injectable hydrogel form with easy application designed for rapid response in emergency room settings.^134^ Furthermore, Leonhardt *et al.* incorporated the functionality of hydrogels as carriers by creating a cyclodextrin polyester hydrogel that can dissolve at the wound site and release chitosan for a hemostatic effect, with demonstrated success and improved functionality *in vivo* compared to pre-existing hemostatic options (Fig. 11).^135^ As a result of these findings among others, research with hydrogels incorporating hemostatic agents and strong adhesive properties is gaining traction, especially in emergency medicine and pre-hospital care settings.

The effectiveness of hydrogels as hemostatic agents varies significantly based on the nature and severity of the wound. However, hemostatic hydrogels still show promise over conventional methods for preventing severe blood loss, such as gauze or tourniquets, as they provide a more supportive environment for wound healing and tissue restoration while also preventing blood loss.^131^ Thus, hydrogels applied for short periods of time in emergency situations may still play a long-term role in chronic wound care when combined with other adaptations to create hospitable environments for long-term tissue repair while providing crucial hemostasis. However, hemostatic hydrogels still face certain limitations that must be addressed. For example, these hydrogels tend to be expensive to manufacture and hard to mass-produce. They also have not yet shown the ability to effectively adhere to skin in all pre-hospital environments and prevent blood loss to the same degree as traditional methods.

### 5.3 Drug delivery agents

Hydrogels are capable of shielding drugs and cells, increasing their therapeutic efficacy. The ability to deliver a variety of cell types and medications to different tissues and organs, while creating a moist environment that promotes favorable conditions for wound healing, is another benefit of hydrogel encapsulation techniques’ versatility. Chronic wounds have an increased risk of infection due to excessive inflammation and prolonged healing. To address these risks, antimicrobial hydrogels have been developed with antibacterial, antiviral, or antifungal properties, depending on the type of infection.^136^

Antibacterial hydrogels are commonly developed to combat infection-causing bacteria in chronic wounds. Specific biocompatible hydrogel dressings with high water content and natural polymers can be used as a matrix for the local administration of drugs released at specific sites to treat chronic wounds.^137^ Antibacterial agents can block bacterial DNA replication or protein synthesis, effectively combating harmful infections and their associated effects.

Hydrogels can naturally exhibit antiviral activity or be engineered to incorporate antiviral agents for enhanced efficacy. For instance, biomaterials that are alginate-based have been shown to have inherent antiviral activities against various types of viruses.^138^ The antiviral activity of hydrogels depends on the bioavailability, solubility, and activity of the antiviral drug incorporated. These properties can be improved by using them in combination with surfactants, polar lipids, or nanoparticles.^137^ In a study reported by Hu *et al.*, an interesting discovery was made in the development of antiviral hydrogels by incorporating drugs with guanine analogues in the presence of metal ions.^139^ These analogs formed hydrogen-bonded quadruplex structures, which induced the formation of supramolecular hydrogels with inherent antiviral activity, which exhibited a controlled release of antiviral drugs in response to a temperature change, which led to the destruction of hydrogen bonds in the hydrogel's structure.

Additionally, some hydrogels have been designed to exhibit antifungal properties that are crucial to prevent fungal infections in chronic wounds. Amphotericin B (AmpB) is a well-known antifungal drug that the FDA has approved the incorporation into hydrogels. Hydrogels that include AmpB are commonly known as amphogels. These amphogels are used in chronic wound healing due to high efficacy against *C. albicans* strains.^140^ High doses of antifungal drugs, like AmpB, that are encapsulated in hydrogels may prove to be toxic to mammalian cells, with the addition of the risk of developing antimicrobial resistance. Researchers have found that to avoid developing antimicrobial resistance, inherent antifungal activity can be provided by the immobilization of biocompatible antifungal peptides into hydrogels.^141^

Hydrogels that incorporate certain inorganic materials exhibit antibacterial properties that can be useful in the wound healing process. Inorganic antibacterial materials mainly include metal and metallic oxide nanoparticles (NPs), such as gold (Au) NPs, silver (Ag) NPs, copper (Cu) NPs, and zinc oxide (ZnO) NPs.^142^ Noble metal ions exhibit long-term inhibition activity on bacterial growth and photo-thermal properties. Highly effective and long-lasting antibacterial activity could be achieved with the combination of both inherent antibacterial activity and heat treatment.^142^ Thus, hydrogels loaded with inorganic NPs could not only enhance the antibacterial properties of a hydrogel, but also maintain antibacterial activity for a longer period of time.^143^ However, the potential toxicity of metal-based materials remains a major concern, particularly when mammalian cells are exposed to them in high concentrations or over extended periods of time.

The incorporation of various therapeutically active substances into hydrogels as drug delivery systems represents an exciting frontier in biomedical research and pharmaceutical development. Stem cells, growth factors, and natural substances are instinctively biocompatible with wound healing and skin regeneration, as these natural therapeutics emphasize the use of biologically derived components to stimulate the body's innate healing processes.^144^ Stem cells often have multi-directional differentiation potential and low immunogenicity, while growth factors are essential for different dynamic stages of wound healing.^61^ Moreover, using natural substances like honey or lemon myrtle oil, traditionally used as a topical paste applied to the skin for wounds, can serve as a low cost component that is naturally antimicrobial and anti-inflammatory.^145^

A list of the diverse types of beneficial agents loaded into hydrogels in drug delivery systems are included below in Table 3.

**Table 3:** Recent agents loaded into hydrogel drug release systems

Loaded agent	Hydrogel polymers	Benefits of agent
**Antibiotics**		
Gentamicin (GEN)^146^	Dextran and polyvinyl alcohol (PVA) based hydrogels. Halloysite-based hydrogels	Provides a positive effect on hydrogel properties like swellability, flexibility, and elasticity; exhibited enhanced healing effect on chronic wounds.
Vancomycin (VAN)^147^	Oligo(poly(ethylene glycol)furmate)/sodium methacrylate (OPF/SMA) charged copolymers biocompatible hydrogel matrices.	Exhibited antimicrobial activity inhibiting the growth of colonies of a clinically derived strain of methicillin-resistant *Staphylococcus aureus*
Ciprofloxacin (CIP)^148^	Keratin-based hydrogels	Inhibited cutaneous wound infection healing without interfering with key aspects of the healing process like tissue deposition and remodeling
Hematoporphyrin^149^	Carbomer (Cu_2_O) – Hematoporphyrin Monomethyl Ether (HMME) hydrogels	Exhibited enhanced antimicrobial ability, excellent biocompatibility, and promoted angiogenesis.
Penicillin^150^	Polymersome hydrogels	Effective against infections Gram-positive cocci, rods and most anaerobes. Known for its effectiveness as an antibiotic and safety.
Moxifloxacin^151^	2-Hydroxyethylmethacrylate (HEMA) based hydrogels	Promotes wound healing without induction to bacterial resistance. Can act as a slow drug delivery system and can overcome the various adverse effects of systemic antibiotic drug delivery.
**Antifungal**		
Amphotericin B (AmpB)^152^	Dextran-based hydrogels	Broad spectrum of antifungal activity, long lasting, marked antifungal efficacy in mice
**Antiviral**		
Penciclovir^153^	Microemulsion-based hydrogels	Effective against herpes simplex virus, varicella zoster virus, Epstein–Barr virus, hepatitis virus and cytomegalovirus.
Entecavir^154^	Supramolecular G-quadruplex hydrogels	First-line treatment option for patients with chronic hepatitis B infection, retains activity against lamivudine-resistant HBV variants
Ganciclovir^155^	Poly(ε-caprolactone)-chitosan polymer hydrogels	Antiviral activity against herpes viral infections, most widely used antiviral to treat cytomegalovirus infections
**Biotherapeutics**		
Bone-marrow derived mesenchymal stem cells (BMSC)^61^	PNIPAM crosslinked with poly(amidoamine) (PAA)	Improved epithelial cell migration and proliferation, collagen deposition, growth factor secretion and wound-healing quality
Mesenchymal stem cells^44^	Myocardium derived extracellular matrix (ECM) hydrogel	Exosome sustained release system promotes angiogenesis, collagen deposition, cell proliferation, and migration, thereby accelerating the wound healing process
Human fibroblast growth factor^62,156^	Polaxam based hydrogel pectin/gum arabic/calcium chloride dihydrate hydrogel	Promoted cell proliferation and migration, rapid regeneration of ECM and angiogenesis; enhanced wound re-epithelialization, collagen deposition, and contraction
Vascular endothelial growth factor (VEGF)^157^	Hyaluronic acid-based hydrogel	Promotion of new blood vessel formation and enhanced supply of oxygen and nutrients to the wound
Lemon myrtle oil (LMO)^145^	Aloe vera-sodium alginate based hydrogel	Improved processability of hydrogel prototypes, increased protein adhesion, enhanced physical properties, and antimicrobial activities against Gram-positive and Gram-negative bacteria and yeasts, and common microbes *Staphylococcus epidermidis* and *Candida albicans*
Honey^158–160^	Poly vinyl alcohol (PVA) hydrogel^158^	Antimicrobial, anti-inflammatory, and antioxidant activity; increased water absorption and swellability, stimulated cell proliferation, supported epithelization, maintained a moist environment to avoid pathogenic infection, and accelerated wound healing in burns
	Cellulose-based hydrogel	
	Chitosan based	
Propolis^161^	Polyacrylamide-methylcellulose hydrogels	Antibacterial properties in the initial stage of the wound repair, antioxidant activity
**Inorganic components**		
Ag nanoparticles^143^	Poly(2-acrylamido-2-methyl-1-propansulfonic acid)	High level of antimicrobial activity without inducing cytotoxicity in mammalian cells at appropriate amounts
Au nanoparticles^162^	Supramolecular hydrogels	Accelerated the healing of diabetic skin wounds, significant levels and increased duration of antimicrobial activity
Cu nanoparticles^163^	Citrate-based hydrogels	Beneficial wound healing of hydrogels in diabetic patients; decreased copper ion toxicity, reduced cell apoptosis, and accelerated wound healing
ZnO nanoparticles^164^	Alginate-based hydrogels	Promoted angiogenesis, leading to faster tissue healing and recovery. Enhance the mechanical properties possessed by natural polymers. Naturally possess antibacterial properties and are photocatalytic in UV light range.

Beyond antimicrobial, antiviral, antifungal, and therapeutic strategies, hydrogels have increasingly been engineered to address chronic wound inflammation through the delivery of immunomodulatory and antiglycation agents. Chronic wounds, particularly diabetic ulcers, are characterized by persistent M1-type macrophage dominance, contributing to sustained inflammation and delayed healing.^165^

Studies have shown that a bilayer alginate hydrogel system encapsulating polyelectrolyte complex nanoparticles (PCNs) loaded with anti-inflammatory cytokines, such as interleukin-10 (IL-10), and angiogenic growth factors can address the challenges of chronic wound healing. The alginate hydrogel is designed to achieve differential degradation, enabling the precise and temporal controlled release of PCNs and loaded agents. The IL-10 released is used to mitigate inflammation, while unsaturated PCNs bind and remove accumulated pro-inflammatory cytokines at the wound site. Then, angiogenic growth factors, such as vascular endothelial growth factor and platelet-derived growth factor, are released, promoting vascularization and vessel maturation.^166^ In addition, hydrogels loaded with macrophage-polarizing agents, such as interleukin-4 (IL-4), interleukin-13 (IL-13), and previously mentioned IL-10, can promote macrophage phenotype switching towards a pro-regenerative M2 state, accelerating resolution of inflammation and improving tissue regeneration.^167^ Furthermore, anti-glycation hydrogels incorporating natural polyphenols like curcumin, epigallocatechin gallate (EGCG), or quercetin have been shown to reduce advanced glycation end-product (AGE) accumulation, a key contributor to oxidative stress and impaired healing in diabetic wounds.^168^

These advanced hydrogel platforms highlight the growing potential of bioactive hydrogel drug delivery systems to not only fight infection but also resolve chronic inflammation a key barrier in chronic wound repair.

## 6 Limitations and future perspectives

This section addresses the challenges related to the mechanical strength, production, and biological compatibility of hydrogels as chronic wound dressings, and explores potential innovations to overcome these limitations. While hydrogels can be engineered to exhibit high mechanical strength, there is typically a trade-off between mechanical robustness and scalable production efficiency.^169^ Given the desired functionality of hydrogels as chronic wound dressings, the ideal hydrogel should balance durability with cost-effective mass production and distribution. This has been particularly achieved through high-density ionic interactions and interpenetrating polymer networks (IPNs), but alternative approaches remain underexplored.^170,171^ Moreover, as wounds can vary greatly across different people based on the type and status of the wound, it may be so that certain aspects of wound healing need to be monitored more during one period and not another. For example, early-stage inflammation is associated with localized temperature increases, but prolonged temperature elevation may indicate infection. A solution for this would be to incorporate sensors with “smart” technologies capable of monitoring wound healing progress, detecting infection, or providing real-time feedback to healthcare providers. Lou *et al.* incorporated an example of this using temperature fluctuations as a wound indicator that would provide early notifications if a wound were infected.^172^ Such a mechanism could be combined with stimuli-sensitive hydrogels to provide optimal wound healing conditions.

Furthermore, incorporating several cross-linkers that have previously been used to generate hydrogels with specific properties, such as glutaraldehyde and epichlorohydrin, have been identified as cytotoxic, which must also be weighed when determining the appropriate hydrogel design for biological applications.^169^ Biocompatible alternatives, like genipin, have been explored for their lower toxicity profiles and suitability in medical applications.^173^ Another issue arises from the replication of specific scaffolds and structures, as well as the maintenance of a particular shape once applied, which can be done successfully in small batches but still faces difficulty in precision on a mass-produced scale.^174^ Injectable hydrogels suffer from similar issues of degradation and inconsistencies in the solidification of hydrogels after injection, primarily due to the variability in environmental conditions (such as pH, temperature, and presence of glucose), which can significantly impact the performance or function of the hydrogel.^175^ There is also a high start-up cost associated with hydrogel fabrication and a lack of systemwide regulation in the process, leading to severe variations in quality and design between manufacturers that will inhibit the rate of production of hydrogels regardless of clinical success.^175^

Lastly, most current hydrogel drug delivery research has been conducted on rat skin. However, rat skin differs significantly from human skin in multiple aspects, particularly in the wound healing process. One such example is how their immune system has many variations in inflammatory cytokine production and immune cell behavior that would affect how they respond to infections, allergens, or injuries compared to humans, leading to deviations in experimental results.^176^ Future recommendations include exploring more suitable wound assessment models for more similar conditions to human skin wounds, and to further clinical trials displaying the efficacies of stimulus-sensitive drug-delivery hydrogels in wound healing.

Previous and current research projects show the promising potential of hydrogels as advanced wound dressings, offering unique properties that distinguish them from conventional dressings commonly used today. However, other directions of hydrogel research have also shown their efficacy in a variety of other applications, including cancer therapy, tissue engineering, nanotechnology, and treatment of osteoarthritis.^177–180^ Given the skin's extensive surface area and its integration with various bodily systems, there is great potential for developing multidisciplinary hydrogels with enhanced healing capabilities. Exploring the possibility further could open new avenues for expanding hydrogel functionality. For example, future hydrogels could combine the drug delivery properties of injectable hydrogels and the incorporation of smart elements to create timed release of photothermal agents for antitumor phototherapy, applications initially popularized in hydrogels for wound dressings.^181^ Furthermore, in the field of nanotechnology, hydrogels have recently gained traction for their conductive and ionic properties that make them ideal for incorporation with materials in the development of wearable technologies. For example, recent advances in MXene-based hydrogels combine the family of 2-D nanomaterials with hydrogels for applications in real-time sensors.^182^ Specifically, the electrical conductivity of MXene materials alongside the biocompatible properties of hydrogels result in ideal characteristics for developing sensors that can detect pressure, strain, and chemical changes related to health, such as in the context of changes indicative of poor wound healing conditions. These scenarios demonstrate the additional efficacy provided by merging the findings of hydrogel research from a variety of unique applications and encourage interconnected and multidisciplinary work as a strong direction for the future of hydrogels.

## 7 Conclusion

Chronic wounds place a significant burden on both healthcare systems and millions of patients worldwide, making the development of more effective treatment strategies imperative. This review has presented a comprehensive overview of chronic wound healing and recent advancements in hydrogel-based dressings designed to enhance their therapeutic efficacy. These innovations have gained increasing attention in recent years due to their ability to create an optimal wound-healing environment, promoting tissue regeneration and repair. Given the complexity of chronic wound healing, internal stimuli-responsive hydrogels have been developed to dynamically respond to environmental cues such as temperature, pH, MMP, ROS levels, or glucose. These changes can serve as indicators of infection or tissue distress, allowing for timely intervention. Additionally, hydrogels have also been modified to release therapeutic agents through drug delivery, providing antimicrobial medication, inorganic compounds, or biotherapeutic substances directly at the wound site. Recent preclinical findings indicate that these innovations can significantly enhance healing outcomes in chronic wounds. However, further studies are needed to optimize their design for large-scale production, ensure long-term biocompatibility, and develop personalized treatments tailored to individual wound conditions.

Moreover, this review emphasizes the growing role of external stimuli-responsive hydrogels, systems that enable active, non-invasive, and precisely controllable therapeutic delivery at chronic wound sites. Compared to conventional drug delivery systems that often rely on passive diffusion or endogenous biological triggers, external stimuli-responsive hydrogels, such as those responsive to mechanical forces, magnetic fields, and ultrasound, offer a greater spatiotemporal control, reduced off-target effects, and adaptability to dynamic wound conditions.^183,184^ These features are particularly important when managing chronic wounds, where timely, localized, and adjustable treatment is essential for overcoming infection and impaired tissue regeneration. As the integration of wearable technologies and smart wound dressings progresses, these external stimuli-responsive systems have the potential to drive the next generation of personalized wound care strategies. This review provides a timely and comprehensive framework to guide future innovation in smart, responsive hydrogel systems targeting chronic wound therapy.

## Conflicts of interest

The authors declare no conflict of interest.

## Data Availability

The data that supports the findings of this study are contained within the article. More information is available on request from the corresponding authors.
